# Genetic variation, recombinant characteristics, and seroprevalence analysis of echovirus 3 causing severe and mild cases of hand, foot, and mouth disease in Guizhou Province

**DOI:** 10.1128/spectrum.03136-25

**Published:** 2026-07-02

**Authors:** Fajin Li, Fei Su, Dan Wang, Fumin Zhang, Jun Guo, Guanghai Yao, Xueqin Ran, Shijun Li, Jiafu Wang

**Affiliations:** 1 College of Life Sciences, Institute of Agro-bioengineering, Key Laboratory of Plant Resource Conservation and Germplasm Innovation in Mountainous Region (Ministry of Education), College of Animal Science, Guizhou University 71206, Guiyang, Guizhou, China; 2 Guizhou Center for Disease Control and Prevention 577390, Guiyang, Guizhou, China; Oklahoma State University College of Veterinary Medicine, Stillwater, Oklahoma, USA; University of Nebraska Medical Center College of Medicine, Omaha, Nebraska, USA; Chinese Academy of Medical Sciences Institute of Laboratory Animal Sciences, Beijing, China

**Keywords:** enterovirus, echovirus 3, hand, foot, and mouth disease, gene recombination, virus transmission, seroprevalence investigation

## Abstract

Echovirus 3 (E-3) has recently been identified as an emerging pathogen associated with both severe and mild cases of hand, foot, and mouth disease (HFMD) in Guizhou Province, China. Although these strains exhibit distinct clinical phenotypes, their underlying molecular mechanisms remain unclear. This study reports the phylogenetic and genetic features of eight E-3 strains from severe and mild HFMD cases and investigates E-3 seroprevalence in Guizhou. Eight shared nucleotide substitutions in the untranslated regions (UTRs) and six shared amino acid substitutions in the open reading frame (ORF) were identified between E-3 strains from severe and mild case groups. Nine variant-specific amino acid mutations were under positive selection and exhibited high variability. Among the 115 nucleotide substitutions in the ORF regions, 31 were specific to the severe case group and 41 to the mild case group. Recombination analysis revealed that the Guizhou E-3 strains underwent complex interserotypic recombination with echovirus 21 (E-21), enterovirus B80 (EV-B80), and echovirus 30 (E-30). E-3 strains from the severe case group demonstrated enhanced replication efficiency, higher viral titers, and greater cytopathogenicity than those from the mild case group. Analysis of 178 samples revealed a seropositivity rate of 52.25% and a geometric mean titer (GMT) of 1:28.23 for E-3, indicating the prevalence of asymptomatic infections in Guizhou Province. All Guizhou E-3 strains exhibited non-temperature-sensitive characteristics, suggesting their potential for transmissibility within the population. This study provides valuable insights for global studies on E-3-related diseases and molecular epidemiology, establishing a foundation for further investigation into the pathogenicity of E-3 variants.

**IMPORTANCE:**

This study is the first report of echovirus 3 (E-3) infections associated with severe hand, foot, and mouth disease (HFMD) cases in Guizhou Province, China. All E-3 strains showed thermotolerance and higher seroprevalence, suggesting potentially enhanced transmissibility. Several shared nucleotide substitutions and amino acid substitutions were identified in Guizhou strains from both severe and mild case groups, indicating that E-3 may have evolved through continuous genetic adaptation, potentially driving the emergence of more transmissible epidemic variants. This study revealed that the E-3 strains in the severe case group exhibited enhanced replication efficiency, higher viral titers, and increased cytopathogenicity *in vitro*, thereby providing evidence to support a potential association between genetic variations and clinical severity. Notably, all E-3-infected patients in the severe case group exhibited more severe clinical manifestations. These findings provide critical updates on the epidemiological trends of enteroviruses associated with HFMD and establish a foundation for a better understanding of genetic diversity, evolutionary patterns, and molecular mechanisms of enterovirus infections.

## INTRODUCTION

Enteroviruses (EVs) are members of the genus *Enterovirus* within the family *Picornaviridae*. The genus *Enterovirus* comprises EV A-L and rhinovirus A-C (1). Among these, EV A-D are groups of viruses associated with a range of human diseases (2). This group includes more than 100 serotypes, including coxsackievirus, poliovirus, echovirus, and several newly identified enteroviruses. Different serotypes of enteroviruses can result in a range of clinical manifestations, including hand, foot, and mouth disease (HFMD), acute flaccid paralysis (AFP), severe respiratory illnesses, myocarditis, aseptic meningitis, encephalitis, and pancreatitis (3–6). Therefore, these viruses have become a significant concern for public health.

Human enteroviruses are small, non-enveloped and single-stranded RNA viruses with a genome of approximately 7.4–7.5 kb, which contains a long open reading frame (ORF) flanked by a 5′-untranslated region (UTR) and a 3′-UTR (7–9). The polyprotein encoded by the ORF can be cleaved into three protein precursors: P1, P2, and P3. These precursors are then processed into four structural proteins (VP1–VP4) as well as seven non-structural proteins (2A–2C and 3A–3D) (2, 3, 10). EV-B comprises numerous serotypes, such as coxsackievirus A9 (CV-A9), CV-B group (serotypes 1–6), echoviruses (serotypes 1–7, 9, 11–21, 24–27, and 29–33), and novel enteroviruses (serotypes 69, 73–75, 77–88, 93, 97–98, 100–101, 106–107, and 110–113) (11–16). A large number of human diseases associated with severe clinical conditions are caused by the EV-B group (5, 14–16). Echovirus 3 (E-3) is a serotype of EV-B that can lead to a range of diseases impacting the human nervous system, including AFP, neonatal hepatitis, paralysis, aseptic meningitis, and other related diseases (17–19). The E-3 prototype strain (AY302553/Morrisey/USA/1951) was first isolated in the United States in 1951 (20–22). E-3 was infrequently detected globally, making it one of the rarest enteroviruses (23–27). This serotype was sporadically reported in the United States in the 1970s and has remained relatively uncommon ever since (24).

E-3 has garnered significant attention due to its association with severe neurological disorders. E-3 strains have primarily been isolated from sewage (27) and patients with aseptic meningitis and AFP (17, 19). Furthermore, there is currently no specific antiviral treatment available for echovirus infections. Consequently, the primary focus of surveillance is on preventing potential complications. However, enterovirus surveillance in Guizhou Province primarily targets AFP cases (with an emphasis on poliovirus) and HFMD cases (focusing on EV-A71 and other enterovirus A species). In addition, the lack of a dedicated surveillance network for E-3 in China has limited the availability of data on its association with clinical manifestations, leaving key aspects such as the disease burden, molecular evolution, and genetic variation of E-3 infections poorly understood. Recently, E-3 strains have been isolated from HFMD cases in Guizhou Province, China, and have been associated with both severe and mild cases. To elucidate the genetic variation associated with clinical manifestations, we performed whole-genome analysis of eight E-3 strains isolated from severe and mild HFMD cases. Our results revealed the potential association between genomic variations in E-3 strains and viral pathogenicity, offering crucial insights into the molecular mechanisms of viral pathogenesis.

## MATERIALS AND METHODS

### Case definition

HFMD was designated as a Category C notifiable infectious disease in May 2008 (28). According to the Chinese guidelines for the diagnosis and treatment of HFMD (2018 edition) (29), suspected cases are characterized by rashes or vesicles on the hands, feet, mouth, or buttocks, as well as scattered herpetic lesions on the oropharynx and oral mucosa, with or without fever. In laboratory testing, enteroviruses can be detected via reverse transcription polymerase chain reaction (RT-PCR) or viral isolation to confirm diagnosis. Patients presenting only with the above manifestations are defined as mild cases. Those with neurological signs including myelitis and meningitis, or cardiopulmonary complications such as pulmonary edema, pulmonary hemorrhage and myocarditis, are classified as severe cases (29).

### Sample collection

Anal and throat swab samples were collected from eight HFMD cases between 1 and 3 years of age. Eight E-6 strains were isolated between 2020 and 2023, including four strains (P0/GZ/CHN/E-3, P17/GZ/CHN/E-3, P22/GZ/CHN/E-3, and P28/GZ/CHN/E-3) isolated from the severe case group and four strains (P30/GZ/CHN/E-3, P37/GZ/CHN/E-3, P40/GZ/CHN/E-3, and P47/GZ/CHN/E-3) isolated from the mild case group (Table S1). Between 2019 and 2022, serum samples were collected from 178 healthy individuals (91 males and 87 females, aged between 6 months and 15 years) from two cities in Guizhou Province: Tongren (*n* = 88) and Zunyi (*n* = 90). Participants were included in the study if they had shown no clinical symptoms (e.g., HFMD) or immunodeficiency disorders for at least 1 month before sampling. Individuals were excluded for recent receipt of relevant vaccines or chronic immunosuppressive therapy. These samples were obtained through the Guizhou Center for Disease Control and Prevention’s (Guizhou CDC’s) HFMD surveillance program.

### Virus isolation and sequencing of the eight strains

Eight specimens were processed according to the standard procedures of specimens and subsequently inoculated into the human rhabdomyosarcoma (RD) cells for virus isolation (29, 30). The RD cell lines were obtained from the Global Poliovirus Specialized Laboratory of the World Health Organization in the United States. After complete EV-like cytopathic effect (CPE) appeared, RD cell cultures infected with enteroviruses were harvested.

Total virus RNA was extracted from the RD cell culture using the viral RNA extraction kit (TIANLONG Co., Ltd, Xi'an, China). RT-PCR was conducted to amplify the partial VP1 region utilizing the PrimeScript One Step RT-PCR Kit Ver.2 (TaKaRa, Dalian, China) with primers 008 and 013 (Forward: GCRTGCAATGAYTTCTCWGT, Reverse: GGIGCRTTICCYTCIGTCCA) (4, 31). The PCR products were purified by the QIAquick Gel Extraction Kit (Qiagen, Hilden, Germany) and subsequently sent to Sangon Biotech Co., Ltd. (Chongqing, China) for DNA sequencing using an automated ABI 3730 DNA sequencer. The EV Genotyping Tool and BLAST server were used to identify the enterovirus serotype (32). The whole-genome sequences of the E-3 strains were amplified using the “primer walking” method as described previously (33). Specific primers were used for RT-PCR to amplify overlapping fragments that encompass the entire genome (Table S2). The 5′ end of the genome was amplified using the E3-0-21S (GCTTTAAAACAGCCTGTGGGT) and E3-570A (AARGAAACACGGACACCCAA) according to the manufacturer’s instructions, and the 3′ end was obtained with the E3-6791S (TTACTTTGTGAGGGGTGGGA) and E3-7428A (TKYACCGAATGSSGAGAATT). The PCR products were purified and sequenced as previously mentioned. The genome sequences detected in this study have been deposited in NCBI GenBank under accession numbers PV137766–PV137773.

### Genetic variance, phylogenetic analysis, and recombination analysis

The Datamonkey online analysis platform was subsequently employed to compute the selection pressure (34). Furthermore, amino acid sites under positive selection pressure were detected by employing the Mixed Effects Model of Evolution (MEME) methodology (*P* < 0.1). The entropy value was analyzed using BioEdit (version 7.0.9.0). An amino acid site with an entropy value greater than 0.6 was defined as highly variable. The three-dimensional protein structure was predicted using the AlphaFold server and subsequently visualized and edited in PyMOL (version 2.5.2).

Nucleotide and amino acid substitution sites identified in the ORF region were compared between the severe and mild case groups using the ClustalW algorithm in MEGA (version 11.0), with the E-3 prototype strain as the reference. The similarity matrix was analyzed with BioEdit (version 7.0.9.0) (35). The phylogenetic trees were constructed utilizing the neighbor-joining method, the Kimura 2-parameter model, and 1,000 bootstrap replicates performed in MEGA 11.0 (36). The BLAST server was used to obtain and filter the full genome sequences based on ≥70% identity. Recombination signals were analyzed using the seven algorithms in RDP5 (version 5.64), with recombination events confirmed only if detected by at least three different methods (*P* < 0.05) (37). The SimPlot software (version 3.5.1) was used to further confirm these assumed recombination events, as previously described (38, 39). The enteroviruses were categorized into four groups. The GZ group served as the query sequence (eight Guizhou E-3 strains); the B80 group (blue line) consisted of three Xinjiang EV-B80 strains; the E21 group (green line) included two Thai E-21 strains; and the E30 group (gray line) comprised four E-30 strains.

### Cytotoxicity and cell viability assessment

RD cells were seeded in 24-well plates (1 × 10⁵ cells per well) and incubated overnight. The E-3 strain was inoculated at a multiplicity of infection (MOI) of 1. After 2 h of adsorption, unbound virions were removed by washing with PBS, and the cells were cultured at 37°C with 5% CO_2_.

Apoptosis in virus-infected RD cells was assessed by the acridine orange/ethidium bromide (AO/EB) double staining method. At 48 h of post-infection, the medium was removed, and the cells were washed with PBS. Then, 200 µL of AO/EB staining solution (10 µg/mL each of AO and EB) was added per well and incubated for 5 min. Images were captured using a fluorescence microscope (20× objective; Nibco, China). Viable cell nuclei exhibited uniform green fluorescence; early apoptotic cells displayed nuclear condensation or fragmentation with yellowish-green to yellow fluorescence; late apoptotic as well as necrotic cells exhibited red fluorescence.

RD cells were seeded in 96-well plates (1 × 10⁴ cells per well) and incubated overnight. The medium was removed, the cells were washed once with PBS, and infected with E-3 strain (MOI = 1) or maintenance medium (cell control). After 1 h at 37°C, the inoculum was discarded and replaced with 2% maintenance medium. Separate wells were used for each time point (6, 12, 24, and 48 h). At each time point, medium was removed and 100 μL of maintenance medium containing 10% CCK-8 reagent was added. Plates were incubated at 37°C in the dark for 2 h, absorbance was measured at 450 nm, and reactions were performed in triplicate. Controls included uninfected cells and blank wells (medium + CCK-8 only). Cell survival rate (%) = [(OD_450_ of the experimental group − OD_450_ of the blank control group)/(OD_450_ of the cell control group − OD_450_ of the blank control group)] × 100%.

Real-time quantitative PCR (qRT-PCR) was employed to evaluate the transcription levels of the VP-1 gene in E-3 strains. The RT-qPCR was performed on a CFX Connect Real-Time System (Bio-Rad Laboratories, Inc., Singapore) using the 2 × Universal Blue SYBR Green qPCR Master Mix (Catalog #: G3326, Servicebio, China) and primers designed based on the VP1 conserved region sequences of E-3. Specific primer pairs for the VP-1 gene (Forward: ACCGAATAGCGCCACAGATT, Reverse: GGGTAAAGTGTGACCACCCA) of the E-3 strain and human GAPDH (Forward: GGAAGCTTGTCATCAATGGAAATC, Reverse: TGATGACCCTTTTGGCTCCC) were designed using Oligo 7.60 software and validated by NCBI Primer-BLAST. All primers were used at 10 pmol/μL. The RT-qPCR assay was carried out in a 20 μL reaction volume, with reactions performed in triplicate. For negative controls, 2 μL of double-distilled H_2_O was used in place of the cDNA template. Amplification efficiency was maintained within the range of 100 ± 10%, as verified by dissociation curve analysis of the PCR products. Relative RNA expression was calculated using the 2^–ΔΔCt^ method with GAPDH as reference.

The virus-infected RD cell monolayers (MOI = 1) were harvested at 6, 12, 24, and 48 h post-infection for the half tissue culture infective dose (TCID_50_) assays in 96-well plates, with viral titers calculated using the Reed-Muench method (3, 40).

### Neutralization assay of E-3

The neutralizing antibody (NtAb) assays were conducted using human RD cell lines, as described in previous studies (40, 41). In the NtAbs test, E-3 strain P0/GZ/CHN/E-3 was selected as the attacking virus because it exhibited the highest titer among the eight E-3 strains. A total of 178 serum samples were inactivated at 56°C in a 5% CO_2_ incubator for 30 min, and then the samples were gradient diluted at 1:4–1:1,024 (1:4, 1:16, 1:64, 1:256, and 1:1,024). The TCID_50_ of 100 (50 μL), serum dilution (50 μL), and 1 × 10^5^ cells/mL human RD cell lines (100 μL) were mixed and then incubated at 36°C in a 5% CO_2_ incubator. In addition, the culture was observed for 7 days using EV-specific CPE, and the highest serum dilution that provided 50% protection for the culture was recorded. If a serum sample with NtAbs titers of ≥1:8 was observed and considered positive, and the geometric mean titers (GMTs) were then calculated (40).

### Assay of temperature sensitivity

Temperature sensitivity is often utilized as an indicator of the attenuation and virulence of enteroviruses *in vitro* (42, 43). In 24-well plates with a monolayer of RD cells, the temperature sensitivity of eight plaque-purified E-3 strains was tested as previously described (3, 44). The inoculation of 50 μL of undiluted virus was inoculated in a 24-well plate. The plate was then placed in two different incubators: one set at the optimal temperature of 36°C and the other at the suboptimal temperature of 39.5°C. After adsorption was performed at 36°C or 39.5°C for 1 h, the unadsorbed viral culture was discarded, and each well was added with 100 μL of cell maintenance medium. The cultures were continuously incubated at temperatures of 36°C or 39.5°C and harvested at six time points: 4, 8, 16, 24, 48, and 72 h. The TCID_50_ was determined using the endpoint dilution method on cultured monolayer RD cells in 96-well plates. All viral titer assays were performed in three independent biological replicates (*n* = 3) for each of the eight Guizhou E-3 strains at every time point (4, 8, 16, 24, 48, and 72 h post-infection) under incubation conditions of 36°C and 39.5°C. The mean viral titer and SD were calculated from the triplicate measurements for each condition, and the mean titers were used to generate the viral growth curves shown in Fig. 7. The difference in mean viral titer between 36°C and 39.5°C was more than 2 log values, and this virus strain was considered temperature sensitive (3, 42, 45).

### Statistical analysis

Selection pressure was estimated using the MEME method implemented on the Datamonkey web server. For each site, MEME estimates one synonymous substitution rate (α) and two distinct nonsynonymous rates (β⁻ and β^+^). A likelihood ratio test (LRT) was conducted between a null model constraining positive selection and an alternative model allowing positive selection. Sites with β^+^ > α and a statistically significant LRT *P*-value were identified as experiencing significant episodic positive selection.

Nucleotide substitution sites in the ORF region were identified by aligning viral sequences from four strains from the severe case group and four strains from the mild case group against the E-3 prototype strain using ClustalW in MEGA (version 11.0). For each identified substitution site, a 2 × 2 contingency table was constructed to compare the distribution of variant nucleotides between the severe and mild case groups. The statistical significance was defined as *P* < 0.05. For nucleotide sites significantly associated with disease severity (*P* < 0.05), the direction of association was defined as the disease group (severe or mild disease group) in which the variant nucleotide (relative to the prototype strain) exhibited a within-group dominant frequency ≥80%. Variants meeting this criterion were classified as associated with the severe or mild disease, respectively.

Differences in cell survival rates, gene expression levels, and virus titers between the severe and mild case groups were analyzed using two-tailed independent-samples *t*-tests at each time point (6, 12, 24, and 48 h) with GraphPad Prism (version 10.6.1). Each group comprised four independent viral strains, with three technical replicates per strain. Data are expressed as mean ± standard deviation (SD). Statistical significance was defined as *P* < 0.05, with ** indicating *P* < 0.01 and *** indicating *P* < 0.001.

## RESULTS

### Clinical characteristics and molecular typing of the eight strains

Eight patients diagnosed with HFMD were under 5 years of age, including four cases with severe symptoms and four with mild symptoms. The four severe cases presented with neurological involvement and high fever (body temperature > 39°C). A rash was present in all cases (Table S1).

**Fig 1:**
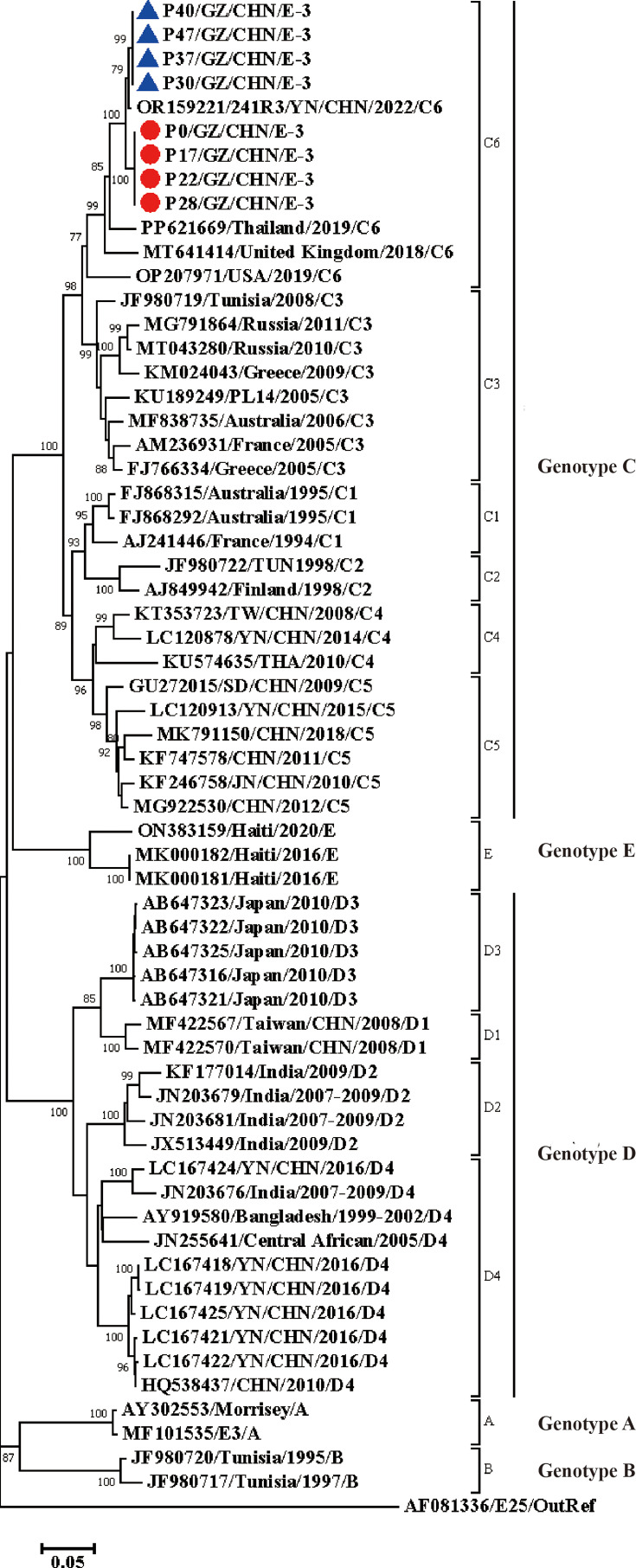
The phylogenetic tree based on the VP1 coding sequences of E-3 obtained from GenBank. The eight E-3 strains in this study were indicated by blue triangles (the four strains from mild cases) and red solid circles (the four strains from severe cases). Bootstrap values >75 were shown and inferred by bootstrapping with 1,000 replicates.

After being cultured in RD cells and harvested upon observation of typical EV-like CPE (3), the eight strains were serotyped by RT-PCR, as previously described (4). The results indicated that the VP1 sequences of Guizhou E-3 strains exhibited 80.4–80.7% nucleotide sequence identity with the E-3 prototype strain. Additionally, the serotype of eight strains was determined to be E-3 based on the VP1 and P1 phylogenetic trees (Fig. 1, 2A and B). Based on the principle of >85% VP1 sequence identity for genotype classification (46), all E-3 strains were identified as subgenotype C6 (Fig. 1), with four strains from the severe case group and four strains from the mild case group forming two separate sub-branches diverging from a common lineage.

**Fig 2:**
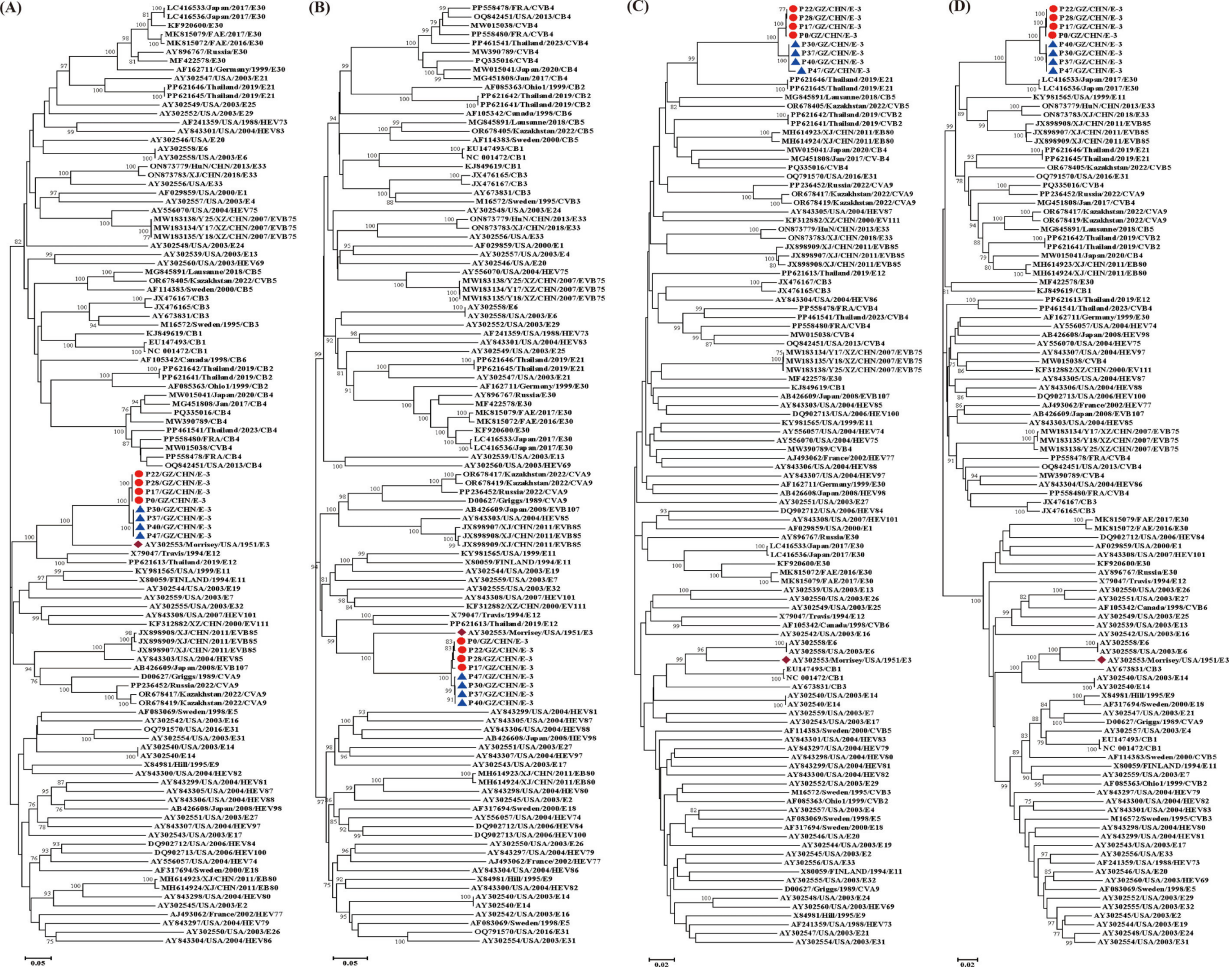
The phylogenetic trees based on the VP1, P1, P2, and P3 sequences of EV-B. The four strains from severe cases were indicated by red solid circles, the four strains from mild cases were indicated by blue triangles, and the E-3 prototype was indicated by a brown diamond. Eight Guizhou E-3 strains and other EV-B strains were analyzed through nucleotide alignment using the neighbor-joining method and the Kimura 2-parameter model conducted in the MEGA 11.0 program. Bootstrap values >75 were shown and inferred by bootstrapping with 1,000 replicates. (**A**) VP1 coding sequences. (**B**) P1 coding sequences. (**C**) P2 coding sequences. (**D**) P3 coding sequences.

### Full-length genomic analysis of the eight E-3 strains

The full-length genomes of the Guizhou E-3 strains were identified to range from 7,428 to 7,430 nucleotides in length. These genomes contained a 5′-UTR of 742 nucleotides, a 3′-UTR of 101–103 nucleotides, and a single ORF of 6,585 nucleotides that encodes 2,195 amino acids. The sequence alignment compared the eight Guizhou E-3 strains with the E-3 prototype strain and revealed that a nucleotide deletion occurred at position 125 and a nucleotide insertion occurred at position 7,338 in all eight E-3 strains.

The total base compositions of the Guizhou E-3 strains were 24.2–24.3% T, 23.1–23.2% C, 27.6–27.8% A, and 24.7–25.0% G. The nucleotide and amino acid identities of the whole genomes were 79.7–79.9% and 95.9–96.3% between the eight Guizhou E-3 strains and the E-3 prototype strain, whereas they showed 82.6–83.0% nucleotide identity and 96.6–96.9% amino acid identity with the strain MK791150/JS/CHN/2018/E-3. In addition, the eight strains exhibited higher similarity to the E-3 prototype strain in the P1 region but greater identity with other EV-B strains in the P2 and P3 regions, suggesting potential recombination events in these regions (Table 1; Table S3; Fig. 2B and D).

**TABLE 1:** Similarity comparisons of nucleotide sequences and deduced amino acid sequences were conducted among eight E-3 strains, the E-3 prototype strain (AY302553), and other enterovirus strains (EV-B and EV-A) in full-length genomic regions^*a*^

Nucleotide identity (%) and amino acid identity (%)						
Region	Comparison with the E-3 prototype strain (%)		Comparison with other EV-B strains (%)		Comparison with other EV-A strains (%)	
	Nucleotide	Amino acid	Nucleotide	Amino acid	Nucleotide	Amino acid
5′-UTR	81.8–82.3	/	55.3–88.9	/	68.1–82.9	/
VP4	77.2–79	94.2–97.1	70–81.9	78.5–97.1	52.8–81.4	24.2–52.2
VP2	78.9–79.3	96.9–98	67.4–73.3	56.9–65.7	50.9–71.6	48.6–80.4
VP3	80.3–80.9	96.6–97	65.1–73.1	71–81.9	49.5–72.4	44.3–80
VP1	80.4–80.7	96.5	57–66.5	54.7–70.1	37.1–62.4	31.2–68.4
2A	79.7	94–94.6	75.7–88.2	90–97.3	64.6–76.8	73.3–91.3
2B	76.7–77.1	92.9	75.7–89.5	92.9–97.9	51.8–78.7	52.5–96.9
2C	80.6–81.7	95.7–97.5	80–89	93.6–98.4	60–85.9	56.2–93.2
3A	78.6–79	93.2–94.3	76.7–91.3	89.8–96.6	52.8–90.2	52.8–96.6
3B	74.2–75.7	95.4	68.1–93.9	81.8–95.4	51.5–87.8	54.5–95.4
3C	78.8–79.2	96.1–96.7	76.6–94.7	95–100	56.8–89.6	53.5–98.3
3D	78.9–79	95.4–95.6	78.2–93.8	94.8–98.4	62.1–85.2	31.4–62.2
3′-UTR	77.3–79.8	/	31.6–90.7	/	34.3–80.7	/

^*a*^ The "/" symbol indicates not applicable. The 5'-UTR and 3'-UTR are non-coding regions that do not translate into amino acid sequences.

To further clarify the evolutionary characteristics of the E-3 strains, 61 complete or nearly complete E-3 genomic sequences (including the 8 Guizhou strains and 53 global reference strains) were downloaded from GenBank and used to construct phylogenetic trees (Fig. S1). All eight Guizhou E-3 strains clustered within genotype C6 in the VP1 region (Fig. S1A), and strains from the mild and severe case groups formed two separate sub-branches diverging from a common lineage. The P1 structural region was relatively conserved, with a branching pattern consistent with the VP1 tree (Fig. S1B). In contrast, the P2 and P3 regions showed greater genetic diversity and divergent topological structures. In the P2 region, E-3 strains from the severe case group clustered with a 2019 Thai strain, while strains from the mild case group clustered with 2022 Yunnan strains (Fig. S1C). In the P3 region, although the overall clustering of strains was similar to the VP1 tree, internal topology and bootstrap support differed markedly from the VP1 and P1 regions (Fig. S1D).

### Phylogenetic analysis of the Guizhou E-3 strains with other EV-B strains

The eight Guizhou strains were clustered together with the prototype strain of E-3 on the VP1 and P1 phylogenetic trees (Fig. 2A and B). Notably, the eight E-3 strains did not cluster with the E-3 prototype strain in the P2 and P3 non-capsid regions, and exhibited a high similarity to other EV-B serotypes, such as the E-21 strains, CV-B2 strains, EV-B80 strains, and E-30 strains (Fig. 2C and D). This finding indicated that recombination may occur between the Guizhou E-3 strains and other EV-B serotypes in these regions. Furthermore, E-3 strains from the severe and mild case groups formed two separate sub-branches in the VP1, P1, P2, and P3 phylogenetic trees, suggesting that the virus might have accumulated specific mutations during its evolution.

### Nucleotide and amino acid variation in the E-3 strains

A total of 115 nucleotide substitutions were identified in the ORF region when comparing E-3 strains from the severe and mild case groups (Table S4). Of these, 31 variant sites were specific to strains in the severe case group, 41 to those in the mild case group, and 43 were shared between the two groups, indicating extensive nucleotide variation in these E-3 strains and distinct mutational patterns associated with disease severity (Table S4). Fifty-eight variation sites showed a statistically significant association with disease severity (*P* < 0.05), with 26 exhibiting a positive association with severe disease and 32 showing a positive association with mild disease, offering valuable insights into potential determinants of viral virulence.

**Fig 3:**
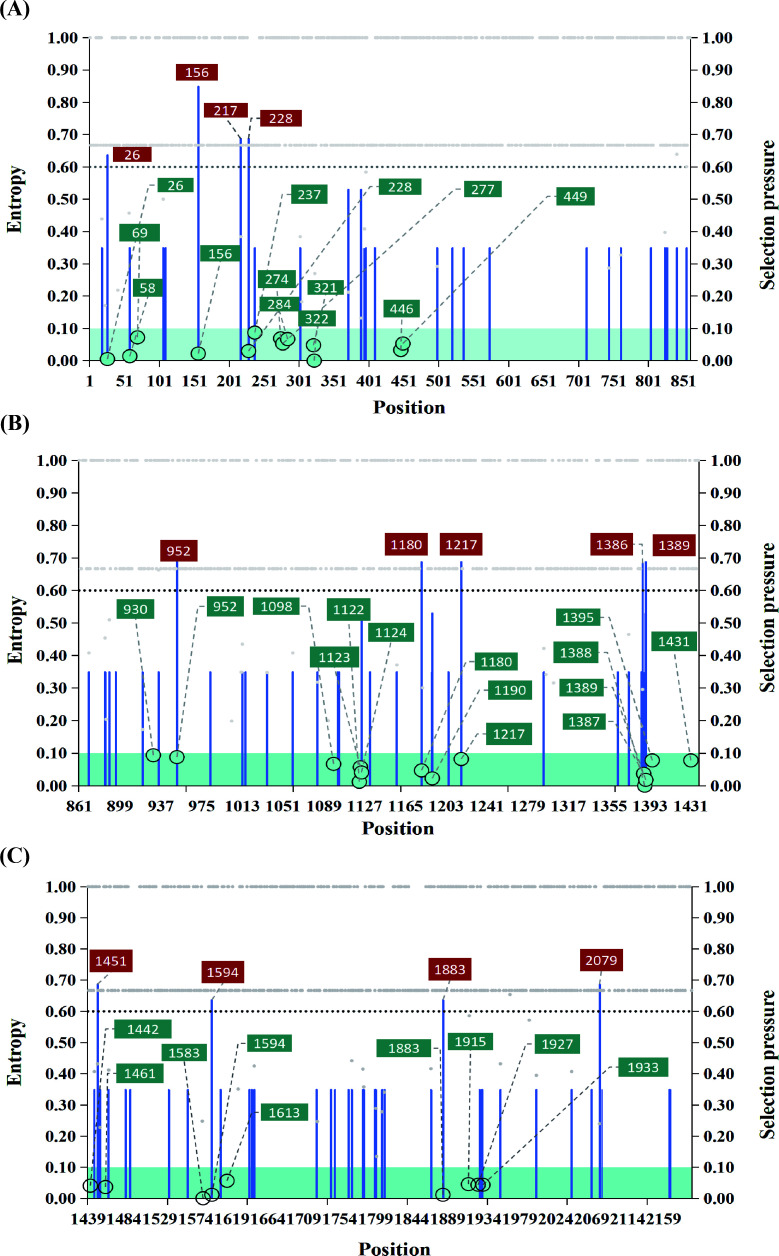
Analysis of amino acid sites under positive selection pressure identified by MEME analysis and sites with high variation identified by entropy value analysis in E-3 strains isolated from mild and severe cases. Green boxes indicate sites under significant positive selection (MEME test, *P* < 0.1). Red boxes highlight sites with high entropy (Shannon entropy > 0.6). Gray dots indicate the remaining sites (shown as background reference). Line height represents the Shannon entropy value (a measure of amino acid diversity). Circles indicate sites under positive selection pressure (*P* < 0.1). All sites are displayed consecutively along the *x*-axis. (**A**) P1 region. (**B**) P2 region. (**C**) P3 region.

**Fig 4:**
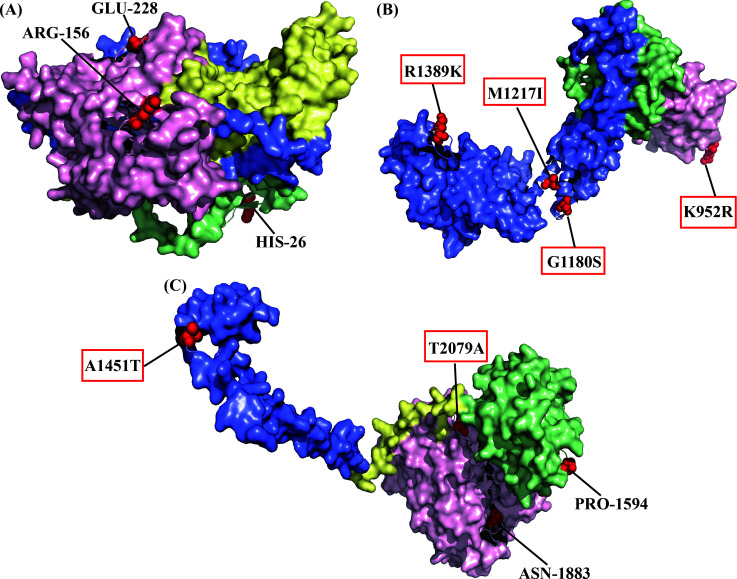
3D structural mapping of key amino acid variations in E-3 virus P1, P2, and P3 polyproteins. (**A**) 3D structure of the P1 capsid polyprotein. VP1 (yellow), VP2 (pink), VP3 (blue), and VP4 (green). (**B**) 3D structure of the P2 non-structural polyprotein. 2A (pink), 2B (green), and 2C (blue). (**C**) 3D structure of the P3 non-structural polyprotein. 3A (blue), 3B (yellow), 3C (green), and 3D (pink). Amino acid sites VP4-26, VP2-156, VP2-228, 2A-952, 2C-1180, 2C-1217, 2C-1389, 3C-1594, and 3D-1883 were under positive selection pressure (MEME test, *P* < 0.1) and highly variable (Shannon entropy > 0.6), and sites 3A-1451 and 3D-2079 were highly variable sites (Shannon entropy > 0.6). Red boxes indicate shared amino acid substitutions between the severe and mild case groups, as well as high-variation or positively selected sites.

Selection pressure and Shannon entropy analysis revealed that several amino acid sites were under positive selection pressure and highly variable (entropy value > 0.6), including sites VP4-26, VP2-156, VP2-228, 2A-952, 2C-1180, 2C-1217, 2C-1389, 3C-1594, and 3D-1883 (Fig. 3 and 4; Tables S5 and S6). Three-dimensional structural mapping showed that these sites were predominantly located on the surface of capsid proteins (such as VP2-156 and VP2-228; Fig. 4A) or within functional domains of non-structural proteins (such as the 2C helicase motif, the 3D polymerase active site; Fig. 4B and C). Interestingly, the shared mutation sites between the mild and severe case groups were observed in the P2 and P3 regions, including sites 2A-952, 2C-1180, 2C-1217, 2C-1389, 3A-1451, and 3D-2079 (Fig. 4B and C; Table S5). Among these, sites 3A-1451 and 3D-2079 were also highly variable (entropy > 0.6). Eight shared nucleotide substitutions were commonly observed in the untranslated regions (UTRs) of E-3 strains from mild and severe case groups (Table S5). Mutations in the 5′-UTR region can impact replication efficiency, cell tropism, and virulence, and specific potential virulence sites have been identified (47–49). Previous studies have suggested that mutations in the P2 and P3 regions can lead to variations in disease severity (50–53). Consistently, our structural localization further shows that these non-structural protein mutations are enriched in functional domains and may affect the clinical severity of E-3 infection.

### Recombinant analysis of the eight strains of E-3

Previous studies have demonstrated that genetic recombination serves as a mechanism of evolution in enteroviruses (54). The breakpoint positions of the recombination analysis in Guizhou E-3 strains were identified at positions 4,034–6,881, 3,752–4,311, 5,203–7,373, and 3,487–4,136 (the site numbers indicate the breakpoint positions of the recombination in the genomic sequence of the Guizhou E-3 strains, Table S3). Some EV-B strains have been identified as the major and minor putative parents, such as CV-B1, CV-B2, CV-B3, CV-B4, EV-B80, E-21, E-30, and EV-B85 (Table S3). Previous research indicated that the Xinjiang EV-B80 strains experienced significant genetic recombination with other EV-B strains (3). Interestingly, in the P2-P3 regions, recombination events were also observed between the Thailand E-21, the Xinjiang EV-B80, the Japan E-30 strains, and the Guizhou E-3 strains (Table S3).

**Fig 5:**
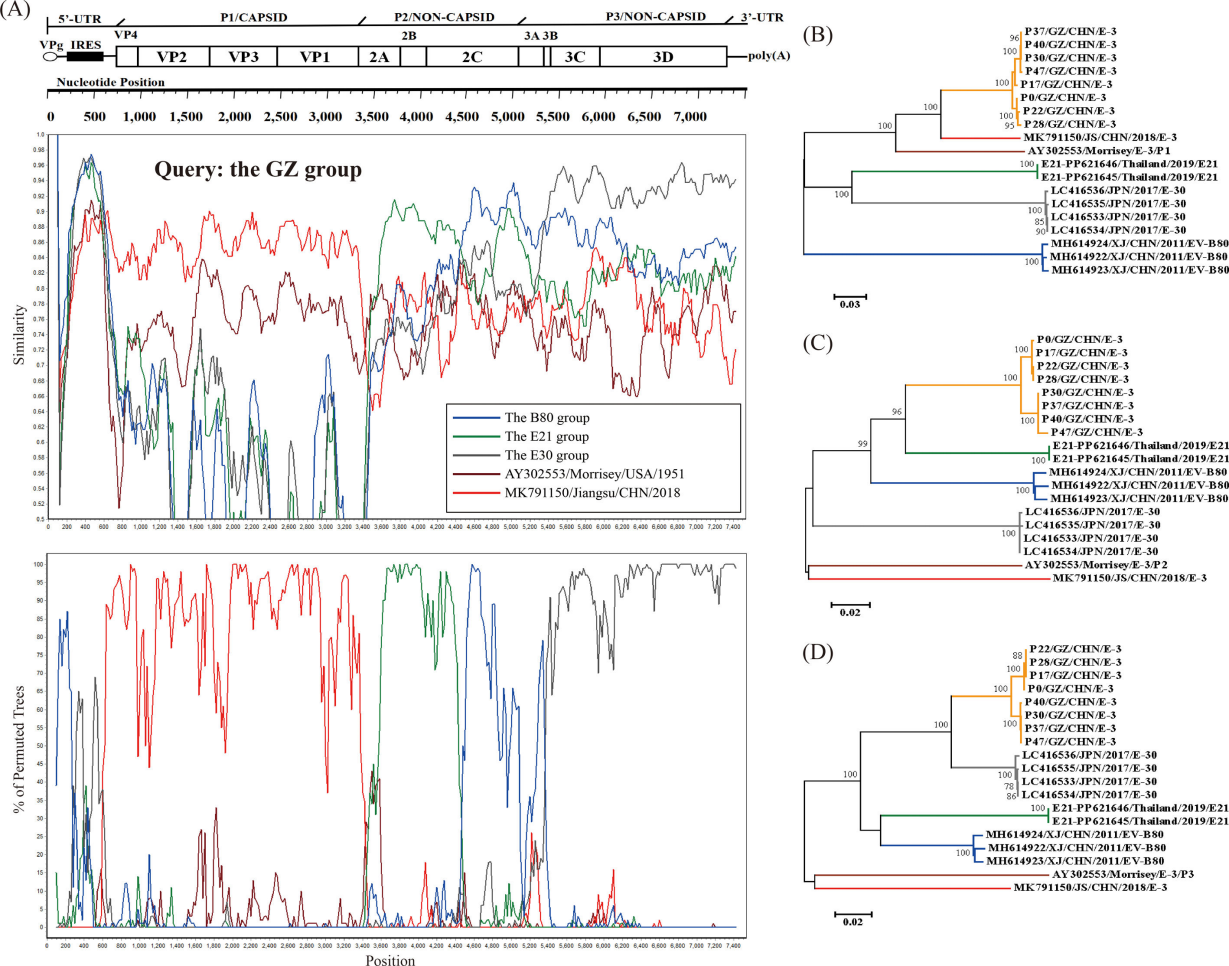
Recombination analyses of the Guizhou E-3 strains with other EV-B strains. Bootstrap values >75 were shown and inferred by bootstrapping with 1,000 replicates. All the tree branches were colored according to the results of the similarity plots and bootscanning analysis. (**A**) The analysis involved similarity and bootscanning among Guizhou E-3, E-21, EV-B80, E-30, Jiangsu E-3 strains, and the E-3 prototype strain to identify potential parental relationships. The GZ group was utilized as the query sequences. (**B**) The phylogenetic tree of the P1 region. (**C**) The phylogenetic tree of the P2 region. (**D**) The phylogenetic tree of the P3 region.

Similarity plots and bootscanning analysis revealed that the GZ group exhibited higher similarity to the Jiangsu E-3 strain in the P1 region (Fig. 5A and B). In the 2A–2C region, the Guizhou E-3 strains were found to recombine with the Thailand E-21 strain (PP621645) from RDP5 (Table S3). The recombination event in the E21 group was identified between positions 3,600 and 4,420, aligning with the findings from RDP5 (Fig. 5A and C; Table S3). Furthermore, the query strains demonstrated a greater likelihood of recombination with the B80 group in the 2C–3A coding region (Fig. 5A and C). In the 3B–3′UTR region, the query strains exhibited a greater likelihood of homology with the E30 group (Fig. 5A and D). The E-21, EV-B80, and E-30 strains were identified as potential donors of the query strains in different regions, as supported by the phylogenetic trees (Fig. 5C and D), which were consistent with the findings from RDP5 (Table S3). The results indicated that the Guizhou strains may have undergone several recombination events with other EV-B viruses.

### Cytotoxicity and cell viability assessment

**Fig 6:**
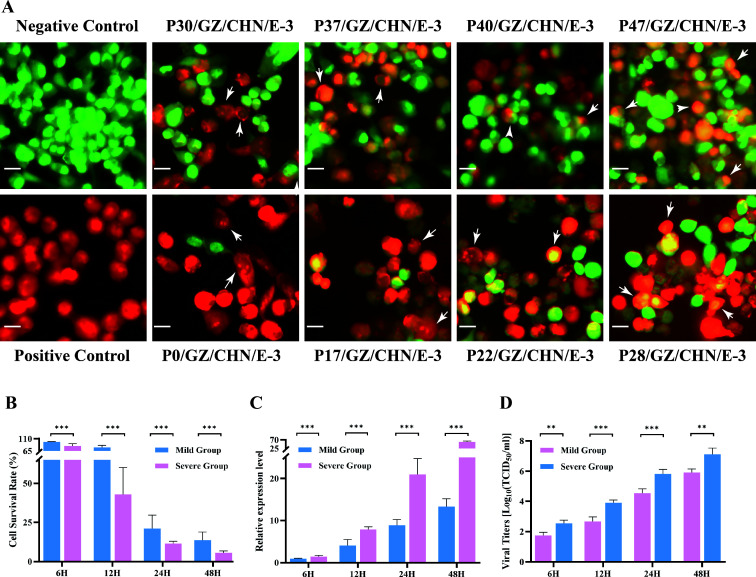
RD cells were infected with the E-3 strains isolated from both mild and severe case groups (MOI = 1). (**A**) AO/EB staining of RD cells at 48 h post-infection with E-3 strains from mild and severe case groups. The P30, P37, P40, and P47 strains were isolated from the mild case group, and the P0, P17, P22, and P28 strains from the severe case group. The nuclei of infected cells exhibited membrane blebbing, condensed chromatin, and fragmented nuclei (indicated by white arrows). Bar = 10 μm. AO penetrates all cells and stains nuclei green, while EB selectively enters cells with compromised membrane integrity, causing red or orange nuclear fluorescence. Consequently, live cells show uniformly green nuclei; early apoptotic cells display nuclear condensation or fragmentation with yellowish-green to yellow fluorescence; late apoptotic as well as necrotic cells exhibit red fluorescence. (**B**) Cell viability of RD cells infected with E-3 strains from mild and severe case groups was assessed by CCK-8. (**C**) Relative RNA expression of E-3 strains from mild and severe case groups was measured by RT-qPCR using *GAPDH* as the internal reference gene. (**D**) Viral titers of the E-3 strains isolated from the mild and severe case groups in RD cells incubated at 37°C were determined using the TCID_50_ assay. The data on viral titers, cell survival rate, and relative expression levels of viral RNA are presented as mean ± SD. Two-tailed independent-samples *t*-tests were used to compare the severe and mild case groups at each time point (**P < 0.01, ***P < 0.001).

AO/EB staining results demonstrated that the E-3 strains isolated from severe cases induced greater pathogenicity and increased cell death due to more extensive cellular damage compared to those from the mild case group at 48 h post-infection, with a significant increase in red fluorescent cells, indicating more pronounced late-stage apoptosis in the strains from the severe case group (Fig. 6A). Cells infected with E-3 strains from the severe case group exhibited significantly lower mean survival rates than those infected with strains from the mild group, as determined by the CCK-8 assay at 6, 12, 24, and 48 h post-infection (Fig. 6B), indicating enhanced cellular pathogenicity of strains isolated from severe cases and consistent with the observed apoptotic activity (Fig. 6A). The mean levels of viral relative RNA were significantly higher in the severe case group than in the mild case group at 6, 12, 24, and 48 h post-infection, as quantified by RT-qPCR, indicating enhanced genomic replication and transcriptional activity (Fig. 6C). Correspondingly, the mean viral titers of the strains from the severe case group were significantly higher at all time points (Fig. 6D). These results demonstrate that E-3 strains from the severe case group have greater capacity for cell invasion, cell destruction, and genomic replication. These findings provide experimental evidence supporting the potential association between virulence differences in E-3 strains and clinical disease severity.

### Seroprevalence of E-3 in Guizhou

Among the 178 serum samples, 93 samples were identified as seropositive (>1:8), with an overall positivity rate of 52.25% and a GMT of 1:28.23. We classified the GMT values into four groups: <1:8, 1:8–1:64, and >1:64 (3, 40), which accounted for 47.75%, 20.79%, and 31.46% of samples, respectively (Table 2). These results indicate that the seroprevalence and GMTs of E-3 were higher than those reported in previous seroepidemiological studies on endemic EV-B serotypes in China, such as EV-B75 and EV-B120 (40, 42). The seroprevalence rate and GMT of E-3 were 54.55% and 1:28.78 in Tongren, and 50% and 1:27.71 in Zunyi, respectively. These findings suggest that the E-3 virus may have caused small-scale outbreaks in these regions, with seroprevalence rates numerically similar to those documented for other HFMD-associated enteroviruses (e.g., EV-A71 and CV-A6) in Chinese populations (55). However, no statistically significant differences were observed in either the seropositivity rate or GMTs of E-3 between the two regions (Seroprevalence rate: *χ*^2^ = 0.368, *P* > 0.05, GMTs: *F* = 0.615, *P* > 0.05).

**TABLE 2:** The compositions of E-3 neutralization antibody titers

Titers groups	Total *n* (%)	Tongren region		Zunyi region	
		Number of samples	Ratio (%)	Number of samples	Ratio (%)
<1:8	85 (47.75)	40	45.45	45	50.00
1:8–1:64	37 (20.79)	22	25.00	15	16.67
>1:64	56 (31.46)	26	29.55	30	33.33
Total	178 (100)	88	100.00	90	100.00

### Temperature sensitivity of the Guizhou E-3 strains

**Fig 7:**
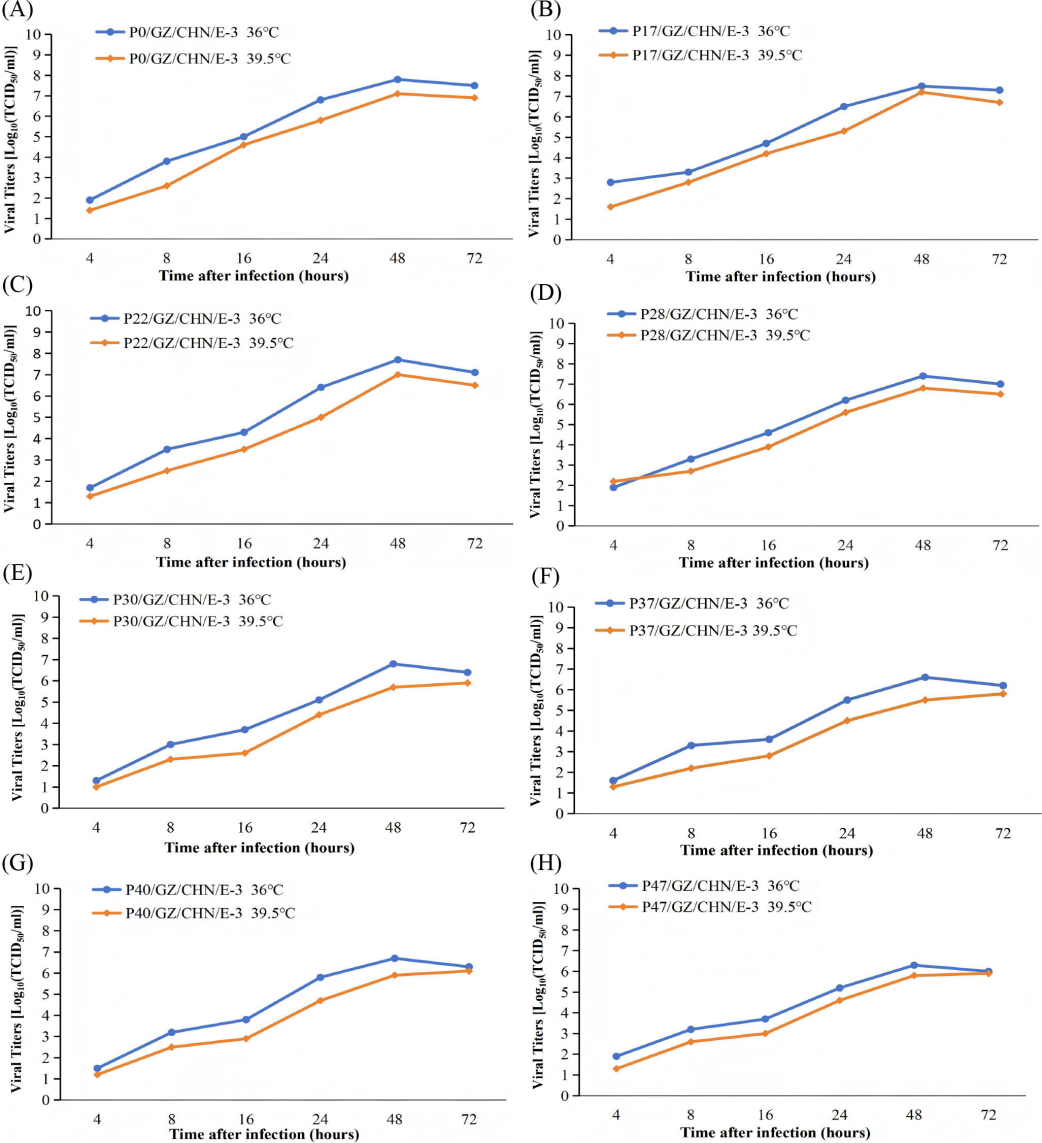
The temperature sensitivity results for the eight Guizhou E-3 strains. The growth trends of the viruses on RD cells at 36°C (blue line) and 39.5°C (orange line) are illustrated in the graph. (**A**) P0/GZ/CHN/E-3 strain. (**B**) P17/GZ/CHN/E-3 strain. (**C**) P22/GZ/CHN/E-3 strain. (**D**) P28/GZ/CHN/E-3 strain. (**E**) P30/GZ/CHN/E-3 strain. (**F**) P37/GZ/CHN/E-3 strain. (**G**) P40/GZ/CHN/E-3 strain. (**H**) P47/GZ/CHN/E-3 strain.

Temperature sensitivity is often utilized as a marker for the attenuation of EV-A71 *in vitro* and is recognized as a significant characteristic of enteroviruses in environmental transmission (56). Although the relationship between temperature sensitivity and viral attenuation may not be direct, it could serve as one of the indicators of enterovirus virulence and its ability to spread. The viral replication capabilities of these eight E-3 strains were compared at temperatures of 36°C and 39.5°C to evaluate their sensitivity to temperature variations. The E-3 strains demonstrated obvious thermotolerance, with a titer difference of less than two logarithms between 36°C and 39.5°C (Fig. 7). This finding indicates that these strains are insensitive to temperature variations.

## DISCUSSION

Since 2013, other enteroviruses have shown a gradual increase in detection frequency and have become the dominant serotypes reported in association with HFMD in China (57, 58), a trend also documented in Guizhou Province (59). Importantly, this apparent shift likely reflects both evolving viral epidemiology and improvements in enterovirus genotyping and diagnostic surveillance. Although HFMD has primarily been associated with EV-A serotypes, EV-B serotypes such as CV-B3, CV-B4, and CV-B5 have also been identified in surveillance, indicating that they may serve as significant HFMD pathogens (60–62). Nationally, the HFMD surveillance network was established in 2008 (28) and has historically focused on EV-A71 and CV-A16, as these serotypes cause the most severe and fatal HFMD cases. EV-B serotypes were not systematically investigated as causative agents of HFMD until approximately 2010–2013 (63, 64), with earlier surveillance focusing almost exclusively on EV-A71 and other EV-A serotypes. Furthermore, enterovirus surveillance in Guizhou has concentrated mainly on AFP (especially poliovirus) and HFMD cases associated with EV-A71 and other EV-A serotypes. Even after broader EV-B monitoring was implemented, E-3 has not been associated with severe HFMD outbreaks, leading to long-term underdetection. Consequently, the relatively low detection rate of E-3 in HFMD patients is likely due to inadequate targeted surveillance. Consistent with limited surveillance, the first nearly full-length E-3 genomes from HFMD patients were reported only in 2022 in Yunnan Province, China (46), and no severe cases of E-3-associated HFMD have been documented globally to date. This study enhances the understanding of E-3 strains isolated from both severe and mild HFMD cases in Guizhou by analyzing their recombination patterns, temperature sensitivity, mutation sites, and seroprevalence, thereby clarifying the epidemic patterns and molecular mechanisms of infection.

Certain serotypes of echovirus (e.g., E-6, E-25, and E-33) have been identified as significant etiological agents associated with HFMD and other neurological disorders (65–67). Novel variants of E-11 genotype D5 were detected in severe and fatal cases in China between 2000 and 2022 (68). This highlights the significance of virological surveillance in the timely detection of highly pathogenic variants. The potential virulence change in E-3 variants from severe cases warrants attention; however, the asymptomatic presentation of enterovirus infections frequently challenges early detection and complicates transmission control. Recent wastewater surveillance in southern Italy since 2022 detected E-11 variants genetically linked to severe neonatal infections, underscoring the value of multi-channel monitoring in preventing enterovirus spread (69). Previous research has indicated that the 5′-UTR of enteroviruses plays a key role in viral virulence (70, 71). The mutation sites T573A and A579G in the 5′-UTR have been associated with enhanced virulence (72) and are located near the shared substitutions (C568T and T583C) identified in strains from both severe and mild cases in this study. Previous evidence has shown that pyrimidine content in this region affects the replication and virulence of enteroviruses (such as CV-B3) (73) and may harbor virulence determinants (such as EV-A71 and poliovirus) (74). Therefore, eight shared mutations in the 5′-UTR of E-3 strains from severe and mild case groups may alter E-3 virulence. The identification of 115 nucleotide substitutions within the ORF, exhibiting distinct patterns between severity groups, highlights extensive genetic diversity associated with clinical outcomes. Notably, 58 sites in E-3 strains were significantly associated with disease severity (*P* < 0.05). These statistically significant variants, particularly the 26 sites positively correlated with severe disease, highlight specific genomic regions that may encode key determinants of viral virulence and thus merit further functional characterization.

Although research on E-3 virulence and clinical manifestations remains limited, studies of enteroviruses such as EV-A71 have associated mutations in the P2 and P3 regions with changes in virulence and disease severity (50–53, 71). This study identified six shared amino acid substitutions in the non-structural proteins between severe and mild case groups. Previous studies have confirmed differences in replication and virulence of enteroviruses isolated from severe and mild cases, differences that are potentially linked to viral pathogenesis (75), with candidate virulence-associated sites for these traits identified in the 2A, 2C, 3A, and 3D regions (43, 51, 52, 76–78). The 2A protein, in particular, plays a critical role in EV-A71 neurovirulence and modulates viral replication and pathogenicity (51). The 2C protein facilitates viral replication by recruiting host reticulon 3 to form a replication complex (79). Notably, in this study, shared mutations in the 2A (K952R) and 2C proteins (G1180S, M1217I, and R1389K) were found to be under positive selection and showed high variability, suggesting that these shared mutations may potentially enhance the virus’s capacity to evade immune recognition and resist clearance by host immune responses.

This study identified a shared mutation at position 3A-1451 (alanine in the E-3 prototype strain and in strains from the mild case group was replaced by threonine in strains from the severe case group), resulting in a shift from a nonpolar to a polar, hydrophilic residue that may alter the dimeric protein’s structure and function. This site high sequence variability, which may facilitate immune evasion, improve RNA replication, increase viral load, and potentially contribute to the development of severe disease. Previous research showed that substituting alanine at 3A-38 (C38A) of CV-B3 reduced replication, suggesting that alanine substitutions in 3A may attenuate viral virulence (80). The 3D-251 (I/V-T) mutation of EV-A71 confers temperature sensitivity, restricting replication and attenuating virulence (81). In this study, the shared 3D-2079 (T2079A) mutation, which is highly variable between severe and mild case groups, may be key to the emergence of the E-3 variant. Although multiple mutations were identified, their biological roles require further experimental validation.

Various studies have confirmed that EV-B serotypes likely underwent intraspecies recombination in non-structural regions, potentially enhancing environmental adaptability and disease severity (3, 40, 82). Several serotypes, including CV-B1, CV-B3, CV-B4, E-21, EV-B80, and E-30, were identified as the major and minor parental strains in this study by RDP5 analysis. Similarity plotting and bootscanning further confirmed recombination events between Guizhou E-3 strains and E-21, EV-B80, and E-30 within the P2 and P3 genomic regions. CV-B3 and CV-B4 were found to be associated with severe neonatal disease (83, 84), and E-30, CV-B1, and CV-B3 were detected in severe cases during a 2014 viral meningitis outbreak in Shandong Province (85). However, the effect of recombination on viral virulence requires further experimental validation.

E-3 strains isolated from the severe case group demonstrated enhanced replication ability, higher viral yields, and greater cytopathogenicity relative to those from the mild case group, as assessed by *in vitro* cellular characteristics. These findings provide supportive evidence for the hypothesis that the identified nucleotide and amino acid substitutions may contribute to viral virulence. Although clinical severity is undoubtedly influenced by host susceptibility factors, these findings demonstrate that differences in intrinsic viral properties provide a key mechanistic basis for disparate disease outcomes. Nevertheless, these correlation analyses represent only preliminary evidence, and the precise role of these variants in virulence determination requires further validation through reverse genetics and animal models. E-3 exhibited higher seroprevalence and GMTs than EV-B75, EV-B120, and EV-B106 (40, 42, 44), consistent with the trends of EV-A71 and CV-A6 in China (55). Although E-3 is rarely reported in HFMD, its widespread circulation in Guizhou indicates substantial transmission potential. In addition, all eight strains were non-temperature-sensitive, suggesting high environmental adaptability and transmissibility, underscoring the need for long-term surveillance.

In summary, we report novel findings on E-3 strains from severe and mild HFMD cases in Guizhou, China. Sequence analysis revealed distinct genetic diversity between E-3 strains from the severe and mild case groups, particularly in the UTRs, 2A, 2C, 3A, and 3D regions, suggesting the accumulation of specific mutations during their divergence from a common ancestor. All eight E-3 strains diverged from the prototype strain in non-structural protein regions and underwent frequent recombination with EV-B serotypes such as E-21, EV-B80, E-30, and CV-B4 within the P2 and P3 regions, reflecting active gene exchange during global circulation. This study demonstrated that the E-3 strain isolated from patients with severe disease exhibited greater replication efficiency, viral titers, and cytopathogenicity *in vitro*, thereby offering support for a correlation between genetic variations and clinical disease severity. Furthermore, seroepidemiological data indicate a relatively high prevalence of E-3 strains in Guizhou, with all strains demonstrating temperature resistance, which may suggest enhanced virulence and transmissibility. Therefore, this serotype may evolve through recombination and gene exchange, potentially leading to wider prevalence. Enhanced surveillance and fundamental research on EV-B serotypes, especially E-3, are essential for understanding pathogenesis and preventing outbreaks. This study provides avenues for further investigation into the relationship between observed genetic variations and the pathogenicity of E-3 strains.

## Supplementary Material

Reviewer comments

## Data Availability

The genome sequences detected in this study have been deposited in NCBI GenBank under accession numbers PV137766–PV137773.

## SUPPLEMENTAL MATERIAL

Fig. S1 spectrum.03136-25-s0001.docx The phylogenetic trees are based on the VP1, P1, P2, and P3 sequences of E-3.

Table S1 spectrum.03136-25-s0002.xls Demographics and clinical characteristics of E-3 positive cases.

Table S2 spectrum.03136-25-s0003.xls PCR and sequencing primer.

Table S3 spectrum.03136-25-s0004.xls The recombination analysis of Guizhou E-3 strains conducted using RDP5 with seven supported methods.

Table S4 spectrum.03136-25-s0005.xls Analysis of nucleotide variation in the ORF region among eight E-3 strains from Guizhou and the E-3 prototype strain.

Table S5 spectrum.03136-25-s0006.xls Nucleotide differences in NTR regions and amino acid variations in coding regions among eight Guizhou E-3 strains and the prototype strain.

Table S6 spectrum.03136-25-s0007.xls Diversifying selection analysis of amino acid sites among eight E-3 strains from Guizhou and the E-3 prototype strain using the MEME method.

OPEN PEER REVIEW reviewer-comments.pdf An accounting of the reviewer comments and feedback.

ASM does not own the copyrights to Supplemental Material that may be linked to, or accessed through, an article. The authors have granted ASM a non-exclusive, world-wide license to publish the Supplemental Material files. Please contact the corresponding author directly for reuse.
