# Supplementary material for: Genetic variation, recombinant characteristics, and seroprevalence analysis of echovirus 3 causing severe and mild cases of hand, foot, and mouth disease in Guizhou Province
Source: Microbiol Spectr. 2026 Jul 2;14(8):e03136-25. doi: 10.1128/spectrum.03136-25 (PMC13436393; doi:10.1128/spectrum.03136-25)
Supplement: Supplementary file 1 [file reviewer-comments.pdf]

AMERICAN SOCIETY FOR MICROBIOLOGY | Microbiology Spectrum

Genetic Variation, Recombinant Characteristics, and Seroprevalence Analysis of Echovirus 3 Causing Severe and Mild Cases of Hand, Foot, and Mouth Disease in Guizhou Province

Fajin Li, Fei Su, Dan Wang, Fumin Zhang, Jun Guo, Guanghai Yao, Xue Ran, Shijun Li, and J Wang

Corresponding Author(s): Shijun Li, Guizhou Provincial Center for Disease Control and Prevention

Review Timeline:

Submission Date:	September 30, 2025
Editorial Decision:	November 17, 2025
Revision Received:	January 22, 2026
Editorial Decision:	February 9, 2026
Revision Received:	April 20, 2026
Accepted:	May 2, 2026

Editor: Clinton Jones

Reviewer(s): Disclosure of reviewer identity is with reference to reviewer comments included in decision letter(s). The following individuals involved in review of your submission have agreed to reveal their identity: Nora M. Chapman (Reviewer #1); lihong zhang (Reviewer #2)

Transaction Report:

(Note: With the exception of the correction of typographical or spelling errors that could be a source of ambiguity, letters and reports are not edited. The original formatting of letters and referee reports may not be reflected in this compilation.)

DOI: https://doi.org/10.1128/spectrum.03136-25

Re: Spectrum03136-25 (Genetic Variation, Recombinant Characteristics, and Seroprevalence Analysis of Echovirus 3 Causing Severe and Mild Cases of Hand, Foot, and Mouth Disease in Guizhou Province)

Dear Dr. Fajin Li:

Thank you for the privilege of reviewing your work. Below you will find my comments, instructions from the Spectrum editorial office, and the reviewer comments.

Please return the manuscript within 60 days; if you cannot complete the modification within this time period, please contact me. If you do not wish to modify the manuscript and prefer to submit it to another journal, notify me immediately so that the manuscript may be formally withdrawn from consideration by Spectrum.

Revision Guidelines
To submit your modified manuscript, log into the submission site at https://spectrum.msubmit.net/cgi-bin/main.plex. Go to Author Tasks and click the appropriate manuscript title to begin. The information you entered when you first submitted the paper will be displayed; update this as necessary. Note the following requirements:

• Upload point-by-point responses to the issues raised by the reviewers in a file named "Response to Reviewers," NOT in your cover letter.
• Upload a compare copy of the manuscript (without figures) as a "Marked-Up Manuscript" file.
• Upload a clean .DOC/.DOCX version of the revised manuscript and remove the previous version.
• Each figure must be uploaded as a separate, editable, high-resolution file (TIFF or EPS preferred), and any multipanel figures must be assembled into one file.
• Any supplemental material intended for posting by ASM should be uploaded with their legends separate from the main manuscript. You can combine all supplemental material into one file (preferred) or split it into a maximum of 10 files with all associated legends included.

For complete guidelines on revision requirements, see our Submission and Review Process webpage. Submission of a paper that does not conform to guidelines may delay acceptance of your manuscript.

Data availability: ASM policy requires that data be available to the public upon online posting of the article, so please verify all links to sequence records, if present, and make sure that each number retrieves the full record of the data. If a new accession number is not linked or a link is broken, provide Spectrum production staff with the correct URL for the record. If the accession numbers for new data are not publicly accessible before the expected online posting of the article, publication may be delayed; please contact production staff (Spectrum@asmusa.org) immediately with the expected release date.

Publication Fees: For information on publication fees and which article types are subject to charges, visit our website. If your manuscript is accepted for publication and any fees apply, you will be contacted separately about payment during the production process; please follow the instructions in that e-mail. Arrangements for payment must be made before your article is published.

ASM Membership: Corresponding authors may join or renew ASM membership to obtain discounts on publication fees. Need to upgrade your membership level? Please contact Customer Service at Service@asmusa.org.

The ASM Journals program strives for constant improvement in our submission and publication process. Please tell us how we can improve your experience by taking this quick Author Survey.

Thank you for submitting your paper to Spectrum.

Sincerely,
Clinton Jones
Editor
Microbiology Spectrum

Reviewer #1 (Comments for the Author):

The authors examined recent isolates of echovirus 3 (E3) from cases of hand foot and mouth disease (HFMD) in the Chinese province Guizhou. E3 is an infrequently isolated enterovirus and has not been associated with commonly associated with HFMD. However, serum from healthy individuals in two cities within the province in the years from 2019 to 2022 had neutralizing titers at levels indicating that E3 was circulating in the community at levels comparable to EV-A71 and CVA6. The authors sequenced 4

E3 isolates from cases of severe HFMD and 4 from cases with mild disease. The authors analyzed the sequences of the isolates to determine the phylogeny and the evidence for recombination with other types of Enterovirus B and found two sets of sequences had a different phylogeny from each other.

These recent E3 isolate sequences are highly related but a number of sites were found in which there was variation between the sequences from the sets of severe and mild disease isolates but the site was conserved within each set of isolates. The authors found 5 such sites in the 5' untranslated region (5'UTR) and 3 in the 3' UTR as well as 6 amino acid variation sites in the open reading frame. The authors have not looked at nucleotide variation in the open reading frame (ORF) despite the fact that there are nucleotide structures such as the oriI or cis-acting replication site within enterovirus ORFs which might well affect virulence. Although the oriI (found in the 2C coding region of enteroviruses) tends to be very well conserved, I did note 15 sites in the nucleotide sequence encoding 2C which had a variation between severe disease viruses and mild disease viruses. The authors discuss the sites with this conservation within each set of sequences which variation between the two sets with the idea that these sites may be the source of the variation in extent of disease. Given that the 4 strains from patients with less severe disease are highly related and so are the 4 strains from patients with severe disease, it will be difficult to find sites which reflect actual virulence with so few isolates. Are these variations found in the databases for earlier isolates of E3? Only testing of variations for ability to cause disease in an animal model or to test whether variations replicate more in restrictive cell lines can provide much evidence for viral determinants of virulence. The authors should note this in the discussion.

The authors have provided a good deal of information on the E3 strain associated recently with HFMD in China and have discussed what can be known about the evolution of the present strains and what might affect the disease severity. As is true for many studies of enterovirus virulence, it is difficult to find the specific determinants without experimentation.

Minor issues:

Lines 46-47: "Among these, EV A-D is a group of viruses associated with a range of human diseases [2]." This should be: "Among these, EV A-D are groups of viruses associated with a range of human diseases [2]."

References 3 and 4 in lines 51 and 9-13 on line 62 don't center on enterovirus disease. There are a number of reviews on this topic that would be better references. On line 55 references 3 and 4 don't center on enterovirus genomes and again there are a number of reviews that cover this. On line 57 a comprehensive review might be: "Baggen J, Thibaut HJ, Strating JRPM, van Kuppeveld FJM. The life cycle of non-polio enteroviruses and how to target it. Nat Rev Microbiol. 2018 Jun;16(6):368-381. doi: 10.1038/s41579-018-0005-4." On Line 60, a good review for this topic is: "Zell R. Picornaviridae-the ever-growing virus family. Arch Virol. 2018 Feb;163(2):299-317. doi: 10.1007/s00705-017-3614-8".

Line 118, primers 008 and 013 are not in reference 6 nor in Table S2. Please identify the primers.

Lines 146-149 refers to Figure 5: please insert (Fig. 5) in this sentence.

In the legend to Figure 3, can you explain the lines versus the circles? Also please number position on X axis as the aa from 1-2915 which would be P1:1-878, P2: 879-1438, P3: 1439-2915 as it is confusing to figure out which sites are what in this figure. I understand that labeling each protein as 1-N would make it difficult to fit. Also, in Table S4, there are a lot of positions with ### rather than a number which again is confusing.

On lines 256-257, Are these numbers the numbering of the prototype strain? And in Table S3 the numbering is XXX (YYY). Is the numbering in the parentheses, that of the prototype or of the specific GZ strain? Please note this in this sentence and in the table.

Reference 5 is not a reference for the sentence on lines 259-260. Please provide the correct reference.

On lines 323-324: Reference 69 is a very limited study from a time period without much experimental data. There are better discussions of this in CVB3 (Phillips L, Tapprich WE. Role of RNA Domain Structure and Orientation in the Coxsackievirus B3 Virulence Phenotype. J Virol. 2023 May 31;97(5):e0044823. doi: 10.1128/jvi.00448-23) and in EV-A71 (Ang PY, Chong CWH, Alonso S. Viral determinants that drive Enterovirus-A71 fitness and virulence. Emerg Microbes Infect. 2021 Dec;10(1):713-724. doi: 10.1080/22221751.2021.1906754).

On lines 331-333: References 45-47 are on mutations in the 5' untranslated region, not the P2 and P3 regions.

On lines 354-355: Reference 55 is a study of an EV-A serotype.

Reference 56 and 77 are identical.

Reviewer #2 (Comments for the Author):

The manuscript titled "Genetic Variation, Recombinant Characteristics, and Seroprevalence Analysis of Echovirus 3 Causing Severe and Mild Cases of Hand, Foot, and Mouth Disease in Guizhou Province"reported the phylogenetic characteristics and genetic evolutionary relationships of eight E-3 strains isolated from severe and mild cases associated with HFMD，and identified eight shared nucleotide substitutions in the non-translated regions (NTRs) and six shared amino acid substitutions in the open reading frame (ORF) between the E-3 strains from severe and mild cases. Meanwhile, complex intraspecies recombination with echovirus 21 (E-21), enterovirus B80 (EV-B80), and echovirus 30 were observed in E-3 strains, the results, if this premise is valid, would establish foundation for further investigation into the pathogenicity of E-3 variants.

However, I have one major concern for the topic of this manuscript.

For viruses, distinguishing between virulent and attenuated strains is a complex and systematic research endeavor. Taking SARS-CoV-2 as an example, we recognize differences in virulence among various subtypes. It is widely accepted that the Beta and Delta subtypes exhibit virulence levels close to the original strain and are considered highly virulent, whereas the Omicron variants are generally less virulent. This conclusion is derived from extensive clinical data analysis and assessments of infectivity

in animal models.
My point is that it is not necessarily true that a strain isolated from a severe case is inherently responsible for causing severe disease, also nor that a strain from a mild case is inherently mild. This is a well-established understanding, which has also been validated in studies of severe and mild hand, foot, and mouth disease. When distinguishing between strains from severe and mild cases based on clinical symptoms in large populations, the boundaries are often blurred. This is because clinical virulence is influenced not only by the viral strain but also by a range of host factors, such as the patient's age, sex, immune status, nutrition and metabolism, timing of hospitalization, and quality of treatment, all of which can impact disease severity.
If the authors intend to analyze nucleotides and recombination patterns from the perspective of "severe" versus "mild" strains, they were suggested first conduct a series of rigorous experiments to confirm the relative virulence of these strains.

Responses to reviewer's comments

Dear Editors and Reviewers:

Thank you for your letter and the comments concerning our manuscript entitled "Genetic Variation, Recombinant Characteristics, and Seroprevalence Analysis of Echovirus 3 Causing Severe and Mild Cases of Hand, Foot, and Mouth Disease in Guizhou Province" (Manuscript Number: Spectrum03136-25R1). Those comments are all valuable and very helpful for revising and improving our paper, and also hold important guiding significance for our research.

Responses to Reviewer #1:

1. Comment

The authors examined recent isolates of echovirus 3 (E3) from cases of hand foot and mouth disease (HFMD) in the Chinese province Guizhou. E3 is an infrequently isolated enterovirus and has not been associated with commonly associated with HFMD. However, serum from healthy individuals in two cities within the province in the years from 2019 to 2022 had neutralizing titers at levels indicating that E3 was circulating in the community at levels comparable to EV-A71 and CVA6. The authors sequenced 4 E3 isolates from cases of severe HFMD and 4 from cases with mild disease. The authors analyzed the sequences of the isolates to determine the phylogeny and the evidence for recombination with other types of Enterovirus B and found two sets of sequences had a different phylogeny from each other.

These recent E3 isolate sequences are highly related but a number of sites were found in which there was variation between the sequences from the sets of severe and mild disease isolates but the site was conserved within each set of isolates. The authors found 5 such sites in the 5' untranslated region (5'UTR) and 3 in the 3' UTR as well as 6 amino acid variation sites in the open reading frame. The authors have not looked at nucleotide variation in the open reading frame (ORF) despite the fact that

there are nucleotide structures such as the oriI or cis-acting replication site within enterovirus ORFs which might well affect virulence. Although the oriI (found in the 2C coding region of enteroviruses) tends to be very well conserved, I did note 15 sites in the nucleotide sequence encoding 2C which had a variation between severe disease viruses and mild disease viruses. The authors discuss the sites with this conservation within each set of sequences which variation between the two sets with the idea that these sites may be the source of the variation in extent of disease. Given that the 4 strains from patients with less severe disease are highly related and so are the 4 strains from patients with severe disease, it will be difficult to find sites which reflect actual virulence with so few isolates. Are these variations found in the databases for earlier isolates of E3? Only testing of variations for ability to cause disease in an animal model or to test whether variations replicate more in restrictive cell lines can provide much evidence for viral determinants of virulence. The authors should note this in the discussion.

The authors have provided a good deal of information on the E3 strain associated recently with HFMD in China and have discussed what can be known about the evolution of the present strains and what might affect the disease severity. As is true for many studies of enterovirus virulence, it is difficult to find the specific determinants without experimentation.

Answer:

Thank you for the valuable suggestion to analyze nucleotide variations within the ORF region. As recommended, we have performed an analysis of ORF nucleotide variations. The key findings are summarized below and detailed in Supplementary Table S4 and in the Abstract, Results and Discussion sections of the manuscript (in blue, lines 21-22, page 1; lines 279-286, page 8; lines 397-402, page 15).

A total of 115 nucleotide substitutions were identified in the ORF regions when comparing severe and mild case groups. Of these, 31 variant sites were specific to the severe case group, 41 to the mild case group, and 43 were shared between the two groups, indicating extensive nucleotide variation in the E-3 strains and divergent

mutational patterns associated with disease severity. The majority were synonymous mutations (101, 87.8%), underscoring the strong evolutionary constraint on the protein sequence. Although non-synonymous variants were less frequent, they may have significant functional implications for protein activity and viral phenotype. Notably, 58 variant sites showed a statistically significant association with disease severity ($p < 0.05$), with 26 exhibiting a positive association with severe cases and 32 showing a negative association with mild cases, highlighting potential determinants of viral virulence.

In direct response to your observation, our analysis confirms multiple variant sites within the 2C region, several of which showed significant association with disease severity and harbored non-synonymous mutations (Table S4). As you hypothesized, these variations could affect their structural stability or protein function, thereby contributing to virulence differences. These findings provide support for in-depth investigation of viral virulence mechanisms.

In conclusion, this nucleotide-level analysis preliminarily elucidates the differential variation spectrum between severe and mild strains and provides a dataset for investigating the molecular mechanisms underlying enterovirus virulence.

We fully agree with your central point: it is difficult to identify specific determinants without experimentation in enterovirus virulence research. This critical perspective has provided essential guidance for refining the direction of this study. In recognition of this complexity, we have avoided using definitive biological descriptors such as "highly virulent strain" or "attenuated strain" throughout this study, and we do not equate isolates from severe cases with "highly virulent strains" in the manuscript. The primary objective of this study is to identify genetic variations and recombination patterns potentially associated with clinical severity through genetic analysis and preliminary in vitro phenotypic comparison of the E-3 strains from severe and mild case groups, thereby providing candidate targets and foundational data for future functional studies. The shared nucleotide and amino acid substitution sites, as well as recombination events identified in the two strain groups, are all based

on sequence differences; their potential contribution to virulence phenotypes requires rigorous experimental validation.

According to your suggestion that "relative virulence must be experimentally verified," we have completed and supplemented the relevant in vitro studies. The in vitro experiments conducted in this study represent an initial exploration based on the aforementioned rationale, aimed at assessing whether these virus strains exhibit quantifiable phenotypic differences under controlled experimental conditions. The supplementary experimental methods and results are summarized below and detailed in Figure 6 and in the Abstract, Methods, Results, and Discussion sections of the manuscript (in blue, lines 24-25, 38-40, page 1; lines 152-190, pages 4-5; lines 324-341, pages 11-13; lines 436-443, page 16).

Eight E-3 strains isolated from patients with severe and mild hand, foot, and mouth disease (HFMD) were selected. Parallel infection experiments were performed in human rhabdomyosarcoma (RD) cells at a strictly consistent multiplicity of infection (MOI = 1) to assess their in vitro biological properties.

Methods

RD cells were seeded in 24-well plates (1×10^5 cells per well) and incubated overnight. The E-3 strain was inoculated at a multiplicity of infection (MOI) of 1. After 2 hours of adsorption, unbound virions were removed by washing with PBS, and the cells were cultured at 37 °C with 5% CO_2.

Apoptosis in virus-infected RD cells was assessed by the acridine orange/ethidium bromide (AO/EB) double staining method. At 48 hours post-infection, the medium was removed and cells were washed with PBS. Then, 200 µL of AO/EB staining solution (10 µg/mL each of AO and EB) was added per well and incubated for 5 minutes. Images were captured using a fluorescence microscope (20× objective; Nibco, China). Viable cell nuclei exhibited uniform green fluorescence; early apoptotic cells displayed nuclear condensation or fragmentation with yellowish-green to yellow fluorescence; late apoptotic as well as necrotic cells exhibited red fluorescence.

RD cells were seeded in 96-well plates (1×10^4 cells per well) and incubated overnight. The medium was removed, cells were washed once with PBS, and infected with E-3 strain (MOI = 1) or maintenance medium (cell control). After 1 h at 37°C, the inoculum was discarded and replaced with 2% maintenance medium. Separate wells were used for each time point (6, 12, 24, and 48 h). At each time point, medium was removed and 100 µL of maintenance medium containing 10% CCK-8 reagent was added. Plates were incubated at 37°C in the dark for 2 h, absorbance was measured at 450 nm, and reactions were performed in triplicate. Controls included uninfected cells and blank wells (medium + CCK-8 only). Cell survival rate (%) = [(OD_{450} of the experimental group - OD_{450} of the blank control group) / (OD_{450} of the cell control group - OD_{450} of the blank control group)] × 100%.

Real-time Quantitative PCR (qRT-PCR) was employed to evaluate the transcription levels of the VP-1 gene in E-3 strains. The RT-qPCR was performed on a CFX Connect™ Real-Time System (Bio-Rad Laboratories, Inc., Singapore) using the 2 × Universal Blue SYBR Green qPCR Master Mix (Catalog #: G3326, Servicebio, China) and primers designed based on the VP1 conserved region sequences of E-3. Specific primer pairs for the VP-1 gene (Forward: ACCGAATAGCGCCACAGATT, Reverse: GGGTAAAGTGTGACCACCCA) of the E-3 strain and human GAPDH (Forward: GGAAGCTTGTCATCAATGGAAATC, Reverse: TGATGACCCTTTTGGCTCCC) were designed using Oligo 7.60 software and validated by NCBI Primer-BLAST. All primers were used at 10 pmol/µL. The RT-qPCR assay was carried out in a 20 µL reaction volume, with reactions performed in triplicate. For negative controls, 2 µL of double-distilled H_2O was used in place of the cDNA template. Amplification efficiency was maintained within the range of 100 ± 10%, as verified by dissociation curve analysis of the PCR products. Relative RNA expression was calculated using the 2–ΔΔCt method with GAPDH as reference.

The virus-infected RD cell monolayers (MOI = 1) were harvested at 6, 12, 24, and 48 hours post-infection for the half tissue culture infective dose ($TCID_{50}$) assays in 96-well plates, with viral titers calculated using the Reed-Muench method.

Differences in cell survival rates, gene expression levels, and virus titers between the mild and severe case groups were analyzed using GraphPad Prism (version 10.6.1), and data are presented as mean ± standard deviation (SD). Statistical significance was defined as $p < 0.05$.

Results

Cytopathic and Apoptotic Effects: Qualitative Assessment

AO/EB staining results demonstrated that the E-3 strains isolated from severe cases induced greater pathogenicity and increased cell death due to more extensive cellular damage compared to those from the mild case group at 48 hours post-infection, with a significant increase in red fluorescent cells, indicating more pronounced late-stage apoptosis in the strains from the severe case group (Fig. 1A).

Quantitative Cytotoxicity Assessment

This quantitative assessment confirmed that cells infected with the severe case-derived E-3 strains exhibited significantly lower mean survival rates compared to those infected with the mild case-derived strains using the CCK-8 assay at each time point (Fig. 1B, Table 1), indicating enhanced cellular pathogenicity associated with the severe isolates, consistent with the observed apoptosis (Fig. 1A).

Viral Replication Capacity Assessment

To evaluate the differences in viral replication between the two E-3 strain groups in vitro, cells were collected at 6, 12, 24, and 48 hours post-infection. Viral relative RNA expression was quantified by RT–qPCR. The mean levels of viral relative RNA in the severe group were higher than those in the mild group, indicating increased genomic replication capacity and transcriptional activity (Fig. 1C, Table 2).

Live Virus Particle Yield ($TCID_{50}$)

The mean viral titers in the severe case group were consistently higher than those in the mild case group across all time points (Fig. 1D, Table 3). This indicates that the viruses isolated from severe cases exhibit enhanced replication capacity and greater efficiency in producing infectious progeny virions.

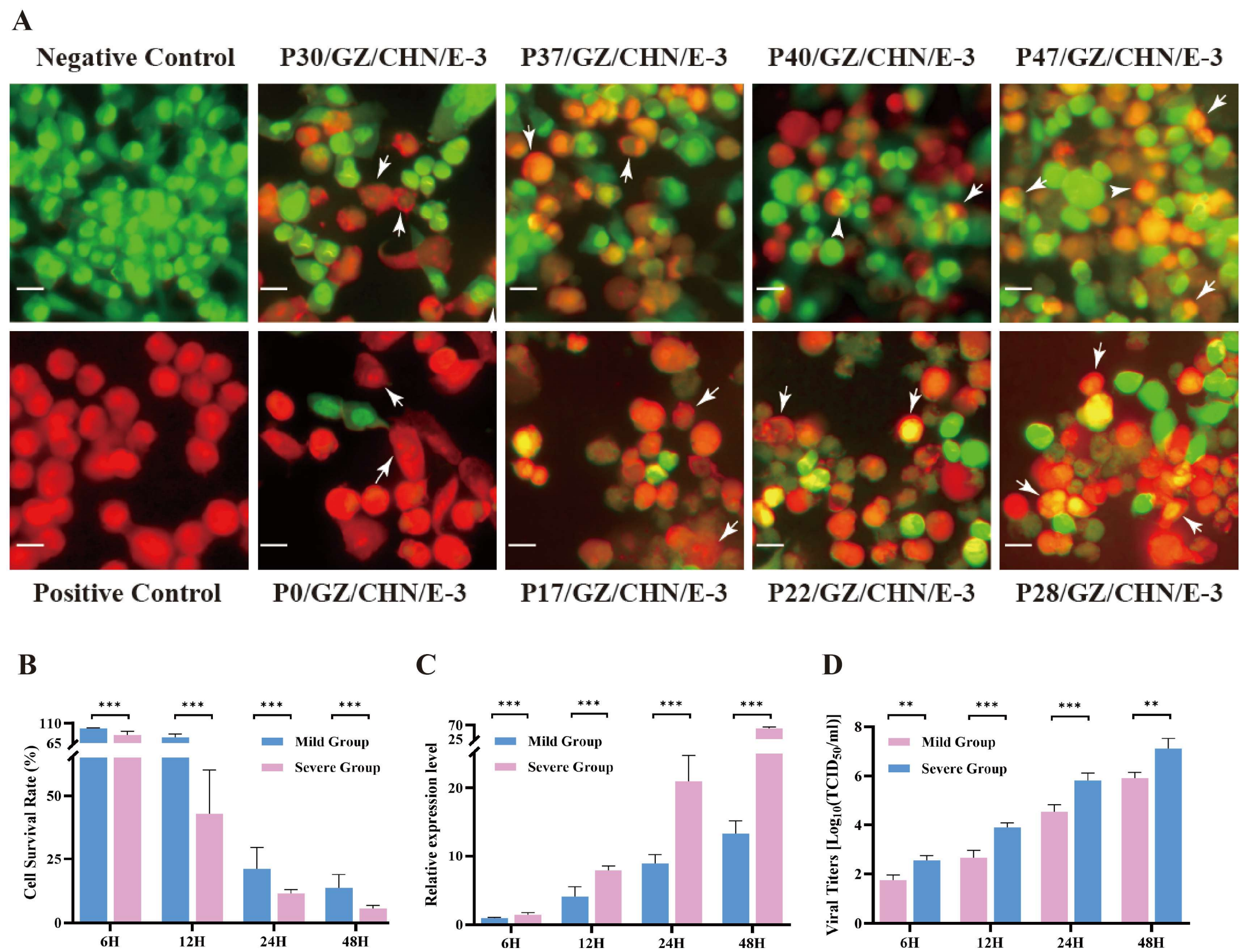

Fig. 1 RD cells were infected with the E-3 strains isolated from both mild and severe clinical case groups (MOI = 1). (A) AO/EB staining of RD cells at 48 hours post-infection with E-3 strains from mild and severe clinical groups. The P30, P37, P40, and P47 strains were isolated from the mild case group, and the P0, P17, P22, and P28 strains from the severe case group. The nuclei of infected cells exhibited membrane blebbing, condensed chromatin, and fragmented nuclei (indicated by white arrow). Bar=10 μm. AO penetrates all cells and stains nuclei green, while EB selectively enters cells with compromised membrane integrity, causing red or orange nuclear fluorescence. Consequently, live cells show uniformly green nuclei; early apoptotic cells displayed nuclear condensation or fragmentation with yellowish-green to yellow fluorescence; late apoptotic as well as necrotic cells exhibited red fluorescence. (B) Cell viability of E-3 strain-infected cells from mild and severe groups was assessed by CCK-8; (C) Relative RNA expression of E-3 strains from mild and severe case groups was measured by RT-qPCR using GAPDH as the internal reference gene. (D) Viral titers of the E-3 strains isolated from the mild and severe case groups in RD cells incubated at 37 °C were determined using the $TCID_{50}$ assay; The data on viral titers, cell survival rate, and relative expression level of virus RNA are presented as mean ± SD for the mild and severe case groups (**$p < 0.01$, ***$p < 0.001$).

Table 1. The cell viability of cells infected with the E-3 strain was compared between mild and severe case groups using the CCK-8 assay at multiple time points.

Time point (h)	Mild case group (n =12)	Severe case group (n =12)	*p* value
6 H	98.265 ± 1.295	83.413 ± 8.566	< 0.001
12 H	78.217 ± 7.571	43.021 ± 17.083	< 0.001
24 H	21.121 ± 8.488	11.405 ± 1.585	< 0.001
48 H	13.667 ± 5.200	5.645 ± 1.153	< 0.001

Data are presented as mean ± SD.

Table 2. The relative RNA expression levels of the E-3 strain were compared between the mild and severe case groups using RT-qPCR at multiple time points.

Time point (h)	Mild case group (n =12)	Severe case group (n =12)	*p* value
6 H	0.949 ± 0.098	1.440±0.314	< 0.001
12 H	4.072±1.422	7.873±0.635	< 0.001
24 H	8.865±1.362	20.948±3.769	< 0.001
48 H	13.291±1.835	59.428±5.196	< 0.001

Data are presented as mean ± SD.

Table 3. The viral titers of the E-3 strain were compared between mild and severe case groups using the $TCID_{50}$ assay at multiple time points.

Time point (h)	Mild case group (n =4)	Severe case group (n =4)	*p* value
6 H	1.75 ± 0.208	2.55 ± 0.208	0.002
12 H	2.68 ± 0.299	3.90 ± 0.183	< 0.001
24 H	4.55 ± 0.289	5.83 ± 0.299	< 0.001
48 H	5.93 ± 0.222	7.13 ± 0.403	0.002

Data are presented as mean ± SD.

In addition to AO/EB staining, we initially attempted to assess apoptosis using the calcein-acetoxymethyl ester (Calcein-AM, 2 μM) and propidium iodide (PI, 6 μM) double fluorescence staining assay (Fig. 2). However, the results were suboptimal for the experimental system. Specifically, we observed that viable cells exhibited only faint green fluorescence (Calcein), which was difficult to distinguish from background, even after systematically testing higher Calcein-AM concentrations (up to 6 μM) and longer incubation times (up to 30–45 minutes) (Fig. 2A–C, white

arrows). We attribute this primarily to the known limitations of the Calcein-AM/PI assay, which is relatively insensitive to early apoptotic cells and whose signal (Calcein) is highly dependent on cellular esterase activity. Calcein-AM enters live cells with intact membranes and is hydrolyzed by intracellular esterases into fluorescent calcein, which emits green fluorescence. In contrast, PI cannot cross intact membranes and only enters cells with compromised membrane integrity—such as those in late apoptosis, necrosis, or after fixation—where it binds to nucleic acids and fluoresces red. Given the need for a method with consistent and clear contrast to reliably quantify apoptotic cells in our model, we ultimately selected the AO/EB staining method for its better contrast and reproducibility.

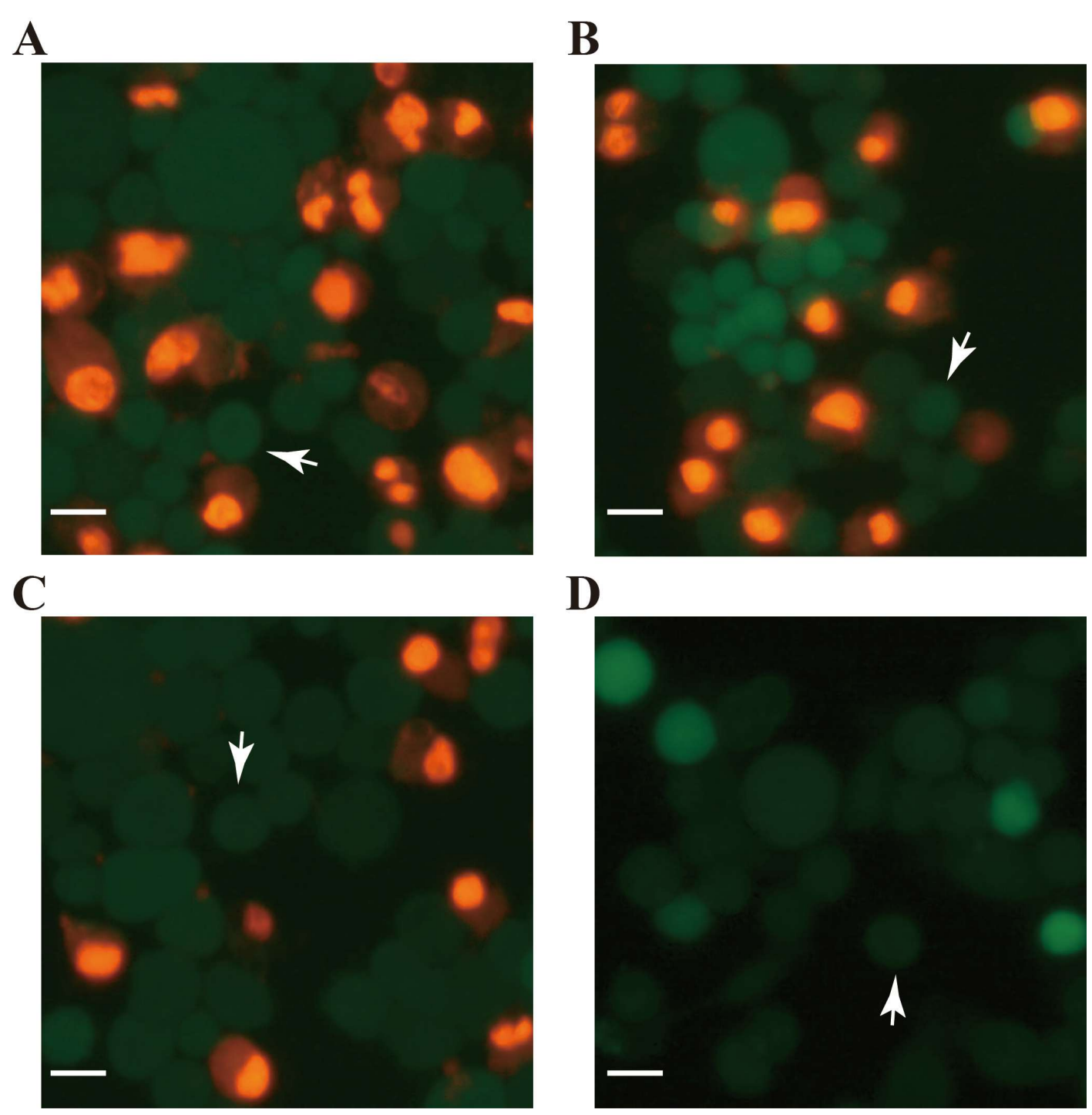

Fig. 2 Calcein Acetoxymethyl Ester (Calcein-AM) / Propidium Iodide (PI) staining of RD cells infected with E-3 strains.Bar=10 μm. (A, B, C) Calcein-AM/PI staining of RD cells infected with E-3 strains. (D) Calcein-AM/PI staining of normal RD cells. Live cells did not exhibit bright green fluorescence, and the green signal was weak and indistinct from the background (indicated by white arrows).

In summary, the isolates from the severe case group exhibit enhanced replication ability, more pronounced cytopathic effects, and increased genomic replication

capacity in the cellular model, providing preliminary support for the potential functional relevance of the observed genetic variations, though these findings are not sufficient to constitute a final conclusion. The in vitro experimental data and genetic analysis results are mutually supportive, establishing a foundation for future functional investigations and enabling the prioritization of specific variant sites and recombination patterns for in-depth research. It should be emphasized that this study is a correlation analysis and preliminary screening research, with its primary contribution residing in the systematic summarization of the genetic features of E-3 in severe and mild case groups and in the establishment of an initial association between genetic variants and in vitro functional phenotypes, thereby providing a clearly defined research direction for future investigations into the core virological determinants underlying the pathogenic mechanism of E-3. In addition, we have carefully ensured objective presentation in our summary, results, and discussion by avoiding absolute simplified terms such as "highly virulent/attenuated strain" or "more/less virulent." It is emphasized that clinical disease severity arises from complex interactions between viral and host factors (e.g., age, immune status), and while enhanced in vitro replication capacity may represent one potential virological basis for severe clinical symptoms, it is by no means the sole determining factor.

2. Comment

Lines 46-47: "Among these, EV A-D is a group of viruses associated with a range of human diseases [2]." This should be: "Among these, EV A-D are groups of viruses associated with a range of human diseases [2]."

Answer:

It has been clarified in the manuscript (in blue, lines 46-47, page 2). We have revised as "Among these, EV A-D are groups of viruses associated with a range of human diseases [2]."

3. Comment

References 3 and 4 in lines 51 and 9-13 on line 62 don't center on enterovirus disease. There are a number of reviews on this topic that would be better references.

Answer:

It has been clarified in the manuscript (lines 51 and 62, page 2).

The review studies have been added to this section as supplementary references (lines 51, page 2).

[1] Liu Y, Maisimu M, Ge Z, Xiao S, Wang H. The Pathogenesis and Virulence of the Major Enterovirus Pathogens Associated with Severe Clinical Manifestations: A Comprehensive Review. Cells. 2025 Oct 17;14(20):1617. doi: 10.3390/cells14201617.

[2] Esposito S, Principi N. Hand, foot and mouth disease: current knowledge on clinical manifestations, epidemiology, aetiology and prevention. Eur J Clin Microbiol Infect Dis. 2018 Mar;37(3):391-398. doi: 10.1007/s10096-018-3206-x.

The originally cited references 9–13 have been updated to the following literature (lines 62, page 2).

[1] Liu Y, Maisimu M, Ge Z, Xiao S, Wang H. The Pathogenesis and Virulence of the Major Enterovirus Pathogens Associated with Severe Clinical Manifestations: A Comprehensive Review. Cells. 2025 Oct 17;14(20):1617. doi: 10.3390/cells14201617.

[2] Zhang M, Wang H, Tang J, He Y, Xiong T, Li W, Qu Y, Mu D. Clinical characteristics of severe neonatal enterovirus infection: a systematic review. BMC Pediatr. 2021 Mar 15;21(1):127. doi: 10.1186/s12887-021-02599-y.

[3] Funaki T, Shoji K. The current status of neonatal echovirus 11 infections in Japan: A comparison to the global situation. J Infect Chemother. 2025 Dec 1;32(1):102879. doi: 10.1016/j.jiac.2025.102879).

4. Comment

On line 55 references 3 and 4 don't center on enterovirus genomes and again there are a number of reviews that cover this.

Answer:

It has been clarified in the manuscript (lines 55, page 2).

The sentence on line 55 originally cited references 5–6, likely as a result of a citation linking error that led to a mismatched reference number. The original references are as follows:

[1] Sean P, Nguyen JH, Semler BL. Altered interactions between stem-loop IV within the 5' noncoding region of coxsackievirus RNA and poly(rC) binding protein 2: effects on IRES-mediated translation and viral infectivity. Virology. 2009 Jun 20;389(1-2):45-58. doi: 10.1016/j.virol.2009.03.012;

[2] Muslin C, Joffret ML, Pelletier I, Blondel B, Delpeyroux F. Evolution and Emergence of Enteroviruses through Intra- and Inter-species Recombination: Plasticity and Phenotypic Impact of Modular Genetic Exchanges in the 5' Untranslated Region. PLoS Pathog. 2015 Nov 12;11(11):e1005266. doi: 10.1371/journal.ppat.1005266.

5. Comment

On line 57 a comprehensive review might be: "Baggen J, Thibaut HJ, Strating JRPM, van Kuppeveld FJM. The life cycle of non-polio enteroviruses and how to target it. Nat Rev Microbiol. 2018 Jun;16(6):368-381. doi: 10.1038/s41579-018-0005-4."

Answer:

It has been clarified in the manuscript (lines 57, page 2).

Reference 10 has been added to the section (Baggen J, Thibaut HJ, Strating JRPM, van Kuppeveld FJM. The life cycle of non-polio enteroviruses and how to target it. Nat Rev Microbiol. 2018 Jun;16(6):368-381. doi: 10.1038/s41579-018-0005-4).

6. Comment

On Line 60, a good review for this topic is: "Zell R. Picornaviridae-the ever-growing virus family. Arch Virol. 2018 Feb;163(2):299-317. doi: 10.1007/s00705-017-3614-8".

Answer:

It has been clarified in the manuscript (lines 60, page 2). Reference 11 has been added to the section (Zell R. Picornaviridae-the ever-growing virus family. Arch Virol. 2018 Feb;163(2):299-317. doi: 10.1007/s00705-017-3614-8).

7. Comment

Line 118, primers 008 and 013 are not in reference 6 nor in Table S2. Please identify the primers.

Answer:

It has been clarified in the manuscript (lines 117–119, page 3).

We have included the relevant information about primers 008 and 013 in the sentence (in blue, lines 117–119, page 3), corrected the original reference number (reference 4), and added new reference 35 as supplements (Hu L, Zhang Y, Hong M, Zhu S, Yan D, Wang D, Li X, Zhu Z, Tsewang, Xu W. Phylogenetic evidence for multiple intertypic recombinations in enterovirus B81 strains isolated in Tibet, China. Sci Rep. 2014 Aug 12;4:6035. doi: 10.1038/srep06035). The sentence on lines 118–119 originally cited reference 4 (Oberste MS, Maher K, Kilpatrick DR, Pallansch MA. Molecular evolution of the human enteroviruses: correlation of serotype with VP1 sequence and application to picornavirus classification. J Virol 1999;73:1941-1948.), likely due to a citation linking error that led to a mismatched reference number.

8. Comment

Lines 146-149 refers to Figure 5: please insert (Fig. 5) in this sentence.

Answer:

It has been clarified in the manuscript (lines 147-150, page 4). Figure 5 (i.e., "(Fig. 5)") has been included in this sentence.

9. Comment

In the legend to Figure 3, can you explain the lines versus the circles? Also please number position on X axis as the aa from 1-2915 which would be P1:1-878, P2: 879-1438, P3: 1439-2915 as it is confusing to figure out which sites are what in this figure. I understand that labeling each protein as 1-N would make it difficult to fit. Also, in Table S4, there are a lot of positions with ### rather than a number which again is confusing.

Answer:

It has been clarified in Figure 3, the Results, and the Discussion of the manuscript (in blue, lines 288-289, 291-292, 298, pages 9-10; lines 412 and 416, page 15; lines 423, page 16). The sequence numbers on the X-axis of Figure 3 have been modified according to the total order of amino acids (Fig. 3). The correspondence between the lines and circles has been clarified in the legend of Figure 3 (in blue). The legend added in Figure 3 is as follows: Lines and circles represent amino acid variation (diversity) and evolutionary driving force (adaptability), respectively. Together, they illustrate the evolutionary landscape of E-3, where positive selection drives variation and increased entropy reflects selective pressure.

In Table S4, the variation site numbers for each nucleotide and amino acid are listed below the corresponding positions (below the corresponding gene). Black dots represent nucleotides or amino acids identical to those in the first strain (P0/GZ/CHN/E-3) at corresponding positions, while non-identical positions are indicated by the actual nucleotide or amino acid. Could it be that discrepancies in file formatting following upload or differences in file versions have resulted in numerous positions marked as ###? Should such issues be confirmed, we will implement appropriate corrections.

The lines and circles in Figure 3 correspond to two aspects: the status of amino

acid site variations (diversity) and the evolutionary driving force (adaptability), respectively. They are complementary and together provide a comprehensive depiction of the evolutionary landscape of E-3.

High entropy/highly variable sites (represented by lines, red boxes): Entropy values exceeding 0.6 are commonly defined as "highly variable", reflecting a diverse array of amino acid types and elevated variation at these sites within the current viral population, as determined by sequence alignment of E-3 isolates (4 from mild cases and 4 from severe cases). This pattern reflects the current status of variation and may arise from processes such as adaptive evolution, neutral drift, or immune selective pressure. Higher entropy values are associated with increased variation at a given site within the viral population, suggesting greater mutational diversity. This elevated variability may reflect viral adaptation to distinct host microenvironments, such as relatively mild immune pressure in individuals with mild disease and stronger immune clearance pressures in severe cases.

Positive selection sites (represented by circles, green boxes): Sites exhibiting ω (dN/dS) > 1 with statistical significance were identified under the phylogenetic model ($p < 0.1$), indicating that amino acid substitutions at these positions are preferentially selected and maintained by natural selection, representing a key evolutionary mechanism that enables the virus to acquire adaptive advantages (such as enhanced infectivity, immune escape, and changes in tissue tropism).

The correspondence between the lines (high-entropy sites with entropy values > 0.6) and the circles (positive selection sites) reveals distinct evolutionary mechanisms underlying amino acid site variation. While positive selection drives increased variability, elevated entropy reflects the effects of such selective pressures.

E-3 needs to adapt to differing host immune pressures, such as elevated antibody levels and heightened inflammatory responses in severe cases during transmission between patients with mild and severe disease. During this process, sites in the P1, P2, and P3 genes associated with viral virulence and immune evasion, such as the 2A protease in the P2 region and the 3C protease in the P3 region, undergo mutations, including amino acid substitutions that enhance protease activity. These variants are

preferentially retained by natural selection (i.e., positive selection), and as the virus undergoes successive transmission cycles, they accumulate progressively, ultimately resulting in high sequence diversity at these positions across different viral strains (entropy value > 0.6).

The entropy value itself reflects only "high variation", with high entropy representing a manifestation of positive selection. In contrast, the detection of positive selection pressure elucidates the underlying evolutionary mechanism driving such variation, indicating not random or neutral mutational drift, but rather the non-random, adaptive accumulation of mutations through natural selection. For instance, overlapping sites in Figure 3 (e.g., Position 1594 and 1883) exhibit both high entropy values (reflecting substantial amino acid divergence between mild and severe strains) and evidence of positive selection (with mutations preserved across lineages), demonstrating that these variants are functionally significant and likely critical for viral adaptation to distinct host environments in mild and severe infections.

A few sites with only high entropy values (without positive selection) indicate that these sites have high diversity in the current strains, but no significant and persistent positive selection signals have been detected. These variations may arise from neutral evolutionary processes or balancing selection. Conversely, sites under positive selection but with low entropy (value < 0.6) reflect ongoing adaptive pressure, where beneficial mutations may have emerged recently or remain at low frequency due to limited transmission or insufficient spread in the population. As a result, these variants do not exhibit high entropy values in the current small sample. Nevertheless, such sites may represent emerging or functionally essential mechanisms of viral adaptability, warranting further longitudinal and functional investigation.

In this study, the simultaneous application of both analytical methods provides a more comprehensive perspective compared to single-method approaches. Rather than merely corresponding to one another, the lines and circles capture complementary aspects of viral evolution through two distinct dimensions of "current status description" and "dynamic inference", which is helpful for precisely identifying the

genetic determinants potentially associated with clinical severity in the P1, P2, and P3 genes of E-3.

10. Comment

On lines 256-257, Are these numbers the numbering of the prototype strain? And in Table S3 the numbering is XXX (YYY). Is the numbering in the parentheses, that of the prototype or of the specific GZ strain? Please note this in this sentence and in the table.

Answer:

It has been clarified in the manuscript (in blue, lines 304–306, page 10) and Table S3.

The strain sources for each site number are clarified in the manuscript as follows (in blue): The site numbers indicate the breakpoint positions of the recombination in the genomic sequence of the Guizhou E-3 strains. The supplementary explanation for Table S3 is as follows: * The numbers outside the parentheses represent the positions of the recombinant breakpoints in the aligned Guizhou sequences that contain gap insertions. The numbers in parentheses represent the recombination breakpoints in the gap-free genome sequence of the Guizhou strains.

The site numbers both inside and outside the parentheses are derived from the analysis results of the input multiple sequence alignment file by RDP5 software. The numbers outside the parentheses (e.g., 4149) represent the positions of the recombinant breakpoints in the aligned Guizhou sequences that contain gap insertions. Specifically, in the multiple sequence alignment of parental and recombinant strains, gaps are inserted to align sequences.

The numbers in parentheses (e.g., 4034) represent the recombination breakpoints in the gap-free genome sequence of the Guizhou strains, representing their actual genomic positions.

11. Comment

Reference 5 is not a reference for the sentence on lines 259-260. Please provide the correct reference.

Answer:

It has been clarified in the manuscript (lines 309, page 10).

The originally cited reference 3 (Han Z, Zhang Y, Huang K, Cui H, Hong M, Tang H, et al. Genetic characterization and molecular epidemiological analysis of novel enterovirus EV-B80 in China. Emerg Microbes Infect. 2018;7:193) is likely attributable to a citation linking error that resulted in an incorrect reference number.

12. Comment

On lines 323-324: Reference 69 is a very limited study from a time period without much experimental data. There are better discussions of this in CVB3 (Phillips L, Tapprich WE. Role of RNA Domain Structure and Orientation in the Coxsackievirus B3 Virulence Phenotype. J Virol. 2023 May 31;97(5):e0044823. doi: 10.1128/jvi.00448-23) and in EV-A71 (Ang PY, Chong CWH, Alonso S. Viral determinants that drive Enterovirus-A71 fitness and virulence. Emerg Microbes Infect. 2021 Dec;10(1):713-724. doi: 10.1080/22221751.2021.1906754).

Answer:

It has been clarified in the manuscript (lines 391, page 15). The originally cited reference 69 has been updated. The updated references are as follows:

[1] Phillips L, Tapprich WE. Role of RNA Domain Structure and Orientation in the Coxsackievirus B3 Virulence Phenotype. J Virol. 2023 May 31;97(5):e0044823. doi: 10.1128/jvi.00448-23.

[2] Ang PY, Chong CWH, Alonso S. Viral determinants that drive Enterovirus-A71 fitness and virulence. Emerg Microbes Infect. 2021 Dec;10(1):713-724. doi: 10.1080/22221751.2021.1906754).

13. Comment

On lines 331-333: References 45-47 are on mutations in the 5' untranslated region, not the P2 and P3 regions.

Answer:

It has been clarified in the manuscript (lines 405, page 15). The cited references 45–47 have been updated to studies linking P2 and P3 region mutations in enteroviruses to changes in virulence and disease severity. The updated references are as follows:

[1] YuF, Xiong D, Zhong Y, Zhang Q-Y, Deng C-L, Zhang Z-R, Wang C, Qiu Y, Ye H-Q, Gong P, Zhang B. Mutation in enterovirus 71 nonstructural protein 3A increases genome replication fidelity and exhibits attenuated virulence in mice. J Virol. 2025 Oct 23;99(10):e0120725. doi: 10.1128/jvi.01207-25.

[2] Ang PY, Chong CWH, Alonso S. Viral determinants that drive Enterovirus-A71 fitness and virulence. Emerg Microbes Infect. 2021 Dec;10(1):713-724. doi: 10.1080/22221751.2021.1906754.

[3] Xu J, Wang F, Zhao D, Liu J, Su H, Wang B. Sequence analysis-based characterization and identification of neurovirulence-associated variants of 36 EV71 strains from China. J Med Virol. 2018 Aug;90(8):1310-1317. doi: 10.1002/jmv.25081.

14. Comment

On lines 354-355: Reference 55 is a study of an EV-A serotype.

Answer:

It has been clarified in the manuscript (lines 428–429, page 16). The cited reference 55 has been updated, and the updated references are as follows:

[1] Li W, Song J, Xu J, Wang H, Duan H, Zhang Y, Xu W, Fan H, Zhang Y. Phylogenetic characteristics and recombination analysis of echovirus 5 associated with severe acute respiratory infection in China. Microbiol Spectr. 2023 Dec 12;11(6):e0171123. doi: 10.1128/spectrum.01711-23.

[2] Li Z, Yang X, Bai R, Li P, Qin Y, Luo M. Circulated echovirus 18 strains in Guangdong Province and worldwide: A novel perspective on genetic diversity and

recombination patterns. Virulence. 2025 Dec;16(1):2534519. doi: 10.1080/21505594.2025.2534519.

15. Comment

Reference 56 and 77 are identical.

Answer:

It has been clarified in the manuscript (lines 611-612, page 20; lines 698-699, page 22).

Reference 47 (Arita M, Shimizu H, Nagata N, Ami Y, Suzaki Y, Sata T, Iwasaki T, Miyamura T. Temperature-sensitive mutants of enterovirus 71 show attenuation in cynomolgus monkeys. J Gen Virol. 2005 May;86(Pt 5):1391-1401. doi: 10.1099/vir.0.80784-0) has been added to this sentence and reference 88 has been updated (Chang GH, Lin L, Luo YJ, Cai LJ, Wu XY, Xu HM, Zhu QY. Sequence analysis of six enterovirus 71 strains with different virulences in humans. Virus Res. 2010 Jul;151(1):66-73. doi: 10.1016/j.virusres.2010.04.001).

Responses to Reviewer #2:

Comment

The manuscript titled "Genetic Variation, Recombinant Characteristics, and Seroprevalence Analysis of Echovirus 3 Causing Severe and Mild Cases of Hand, Foot, and Mouth Disease in Guizhou Province"reported the phylogenetic characteristics and genetic evolutionary relationships of eight E-3 strains isolated from severe and mild cases associated with HFMD， and identified eight shared nucleotide substitutions in the non-translated regions (NTRs) and six shared amino acid substitutions in the open reading frame (ORF) between the E-3 strains from severe and mild cases. Meanwhile, complex intraspecies recombination with echovirus 21 (E-21), enterovirus B80 (EV-B80), and echovirus 30 were observed in E-3 strains, the results, if this premise is valid, would establish foundation for further investigation into the pathogenicity of E-3 variants.

However, I have one major concern for the topic of this manuscript.

For viruses, distinguishing between virulent and attenuated strains is a complex and systematic research endeavor. Taking SARS-CoV-2 as an example, we recognize differences in virulence among various subtypes. It is widely accepted that the Beta and Delta subtypes exhibit virulence levels close to the original strain and are considered highly virulent, whereas the Omicron variants are generally less virulent. This conclusion is derived from extensive clinical data analysis and assessments of infectivity in animal models.

My point is that it is not necessarily true that a strain isolated from a severe case is inherently responsible for causing severe disease, also nor that a strain from a mild case is inherently mild. This is a well-established understanding, which has also been validated in studies of severe and mild hand, foot, and mouth disease. When distinguishing between strains from severe and mild cases based on clinical symptoms in large populations, the boundaries are often blurred. This is because clinical virulence is influenced not only by the viral strain but also by a range of host factors, such as the patient's age, sex, immune status, nutrition and metabolism, timing of hospitalization, and quality of treatment, all of which can impact disease severity. If the authors intend to analyze nucleotides and recombination patterns from the perspective of "severe" versus "mild" strains, they were suggested first conduct a series of rigorous experiments to confirm the relative virulence of these strains.

Answer:

We gratefully acknowledge the reviewer for the valuable feedback. We fully agree with your central point: viral virulence cannot be inferred from clinical categorization or genetic sequence variations alone, as disease severity is determined by multiple factors, including the host's immune status, age, and underlying diseases; it must be validated by a series of rigorous experiments. In recognition of this complexity, we have avoided using definitive biological descriptors such as "highly virulent strain" or "attenuated strain" throughout this study, and we do not equate isolates from severe cases with "highly virulent strains" in the manuscript. The primary objective of this study is to identify genetic variations and recombination

patterns potentially associated with clinical severity through genetic analysis and preliminary in vitro phenotypic comparison of the E-3 strains from severe and mild case groups, thereby providing candidate targets and foundational data for future functional studies. The shared nucleotide and amino acid substitution sites, as well as recombination events identified in the two strain groups, are all based on sequence differences; their potential contribution to virulence phenotypes requires rigorous experimental validation.

In response to your key recommendation that "a series of rigorous experiments be conducted to confirm the relative virulence of these strains," we have completed and supplemented the relevant in vitro studies. The in vitro experiments conducted in this study represent an initial exploration based on the aforementioned rationale, aimed at assessing whether these virus strains exhibit quantifiable phenotypic differences under controlled experimental conditions. The supplementary experimental methods and results are summarized below and detailed in Figure 6 and in the Abstract, Methods, Results, and Discussion sections of the manuscript (in blue, lines 24-25, 38-40, page 1; lines 152-190, pages 4-5; lines 324-341, pages 11-13; lines 436-443, page 16).

Eight E-3 strains isolated from patients with severe and mild hand, foot, and mouth disease (HFMD) were selected. Parallel infection experiments were performed in human rhabdomyosarcoma (RD) cells at a strictly consistent multiplicity of infection (MOI = 1) to assess their in vitro biological properties.

Methods

RD cells were seeded in 24-well plates (1×10^5 cells per well) and incubated overnight. The E-3 strain was inoculated at a multiplicity of infection (MOI) of 1. After 2 hours of adsorption, unbound virions were removed by washing with PBS, and the cells were cultured at 37 °C with 5% CO_2.

Apoptosis in virus-infected RD cells was assessed by the acridine orange/ethidium bromide (AO/EB) double staining method. At 48 hours post-infection, the medium was removed and cells were washed with PBS. Then, 200 μL of AO/EB staining solution (10 μg/mL each of AO and EB) was added per well

and incubated for 5 minutes. Images were captured using a fluorescence microscope (20× objective; Nibco, China). Viable cell nuclei exhibited uniform green fluorescence; early apoptotic cells displayed nuclear condensation or fragmentation with yellowish-green to yellow fluorescence; late apoptotic as well as necrotic cells exhibited red fluorescence.

RD cells were seeded in 96-well plates (1×10^4 cells per well) and incubated overnight. The medium was removed, cells were washed once with PBS, and infected with E-3 strain (MOI = 1) or maintenance medium (cell control). After 1 h at 37°C, the inoculum was discarded and replaced with 2% maintenance medium. Separate wells were used for each time point (6, 12, 24, and 48 h). At each time point, medium was removed and 100 μL of maintenance medium containing 10% CCK-8 reagent was added. Plates were incubated at 37°C in the dark for 2 h, absorbance was measured at 450 nm, and reactions were performed in triplicate. Controls included uninfected cells and blank wells (medium + CCK-8 only). Cell survival rate (%) = [(OD_{450} of the experimental group - OD_{450} of the blank control group) / (OD_{450} of the cell control group - OD_{450} of the blank control group)] × 100%.

Real-time Quantitative PCR (qRT-PCR) was employed to evaluate the transcription levels of the VP-1 gene in E-3 strains. The RT-qPCR was performed on a CFX Connect™ Real-Time System (Bio-Rad Laboratories, Inc., Singapore) using the 2 × Universal Blue SYBR Green qPCR Master Mix (Catalog #: G3326, Servicebio, China) and primers designed based on the VP1 conserved region sequences of E-3. Specific primer pairs for the VP-1 gene (Forward: ACCGAATAGCGCCACAGATT, Reverse: GGGTAAAGTGTGACCACCCA) of the E-3 strain and human GAPDH (Forward: GGAAGCTTGTCATCAATGGAAATC, Reverse: TGATGACCCTTTTGGCTCCC) were designed using Oligo 7.60 software and validated by NCBI Primer-BLAST. All primers were used at 10 pmol/μL. The RT-qPCR assay was carried out in a 20 μL reaction volume, with reactions performed in triplicate. For negative controls, 2 μL of double-distilled H_2O was used in place of the cDNA template. Amplification efficiency was maintained within the range of 100

± 10%, as verified by dissociation curve analysis of the PCR products. Relative RNA expression was calculated using the 2–ΔΔCt method with GAPDH as reference.

The virus-infected RD cell monolayers (MOI = 1) were harvested at 6, 12, 24, and 48 hours post-infection for the half tissue culture infective dose ($TCID_{50}$) assays in 96-well plates, with viral titers calculated using the Reed-Muench method.

Differences in cell survival rates, gene expression levels, and virus titers between the mild and severe case groups were analyzed using GraphPad Prism (version 10.6.1), and data are presented as mean ± standard deviation (SD). Statistical significance was defined as $p < 0.05$.

Results

Cytopathic and Apoptotic Effects: Qualitative Assessment

AO/EB staining results demonstrated that the E-3 strains isolated from severe cases induced greater pathogenicity and increased cell death due to more extensive cellular damage compared to those from the mild case group at 48 hours post-infection, with a significant increase in red fluorescent cells, indicating more pronounced late-stage apoptosis in the strains from the severe case group (Fig. 1A). AO can penetrate all cells and stain the nuclei green, whereas EB selectively enters cells with compromised membrane integrity, resulting in red or orange nuclear fluorescence. Consequently, Viable cell nuclei exhibited uniform green fluorescence; early apoptotic cells displayed nuclear condensation or fragmentation with yellowish-green to yellow fluorescence; late apoptotic cells exhibit condensed or fragmented chromatin stained red or orange; late apoptotic as well as necrotic cells exhibited red fluorescence.

Quantitative Cytotoxicity Assessment

To assess the effect of the E-3 strains isolated from patients with severe and mild disease on cell viability, cell viability was measured using the CCK-8 assay at 6, 12, 24, and 48 hours post-infection. These results showed that cells infected with the severe case-derived E-3 strains exhibited significantly lower mean survival rates compared to those infected with the mild case-derived strains at each time point (Fig.

1B, Table 1), indicating enhanced cellular pathogenicity associated with the severe isolates, consistent with the observed apoptosis (Fig. 1A).

Viral Replication Capacity Assessment

To evaluate the differences in viral replication between the two E-3 strain groups in vitro, cells were collected at 6, 12, 24, and 48 hours post-infection. Viral relative RNA expression was quantified by RT–qPCR. The mean levels of viral relative RNA in the severe group were higher than those in the mild group, indicating increased genomic replication capacity and transcriptional activity (Fig. 1C, Table 2).

Live Virus Particle Yield ($TCID_{50}$)

The mean viral titers in the severe case group were consistently higher than those in the mild case group across all time points (Fig. 1D, Table 3). This indicates that the viruses isolated from severe cases exhibit enhanced replication capacity and greater efficiency in producing infectious progeny virions.

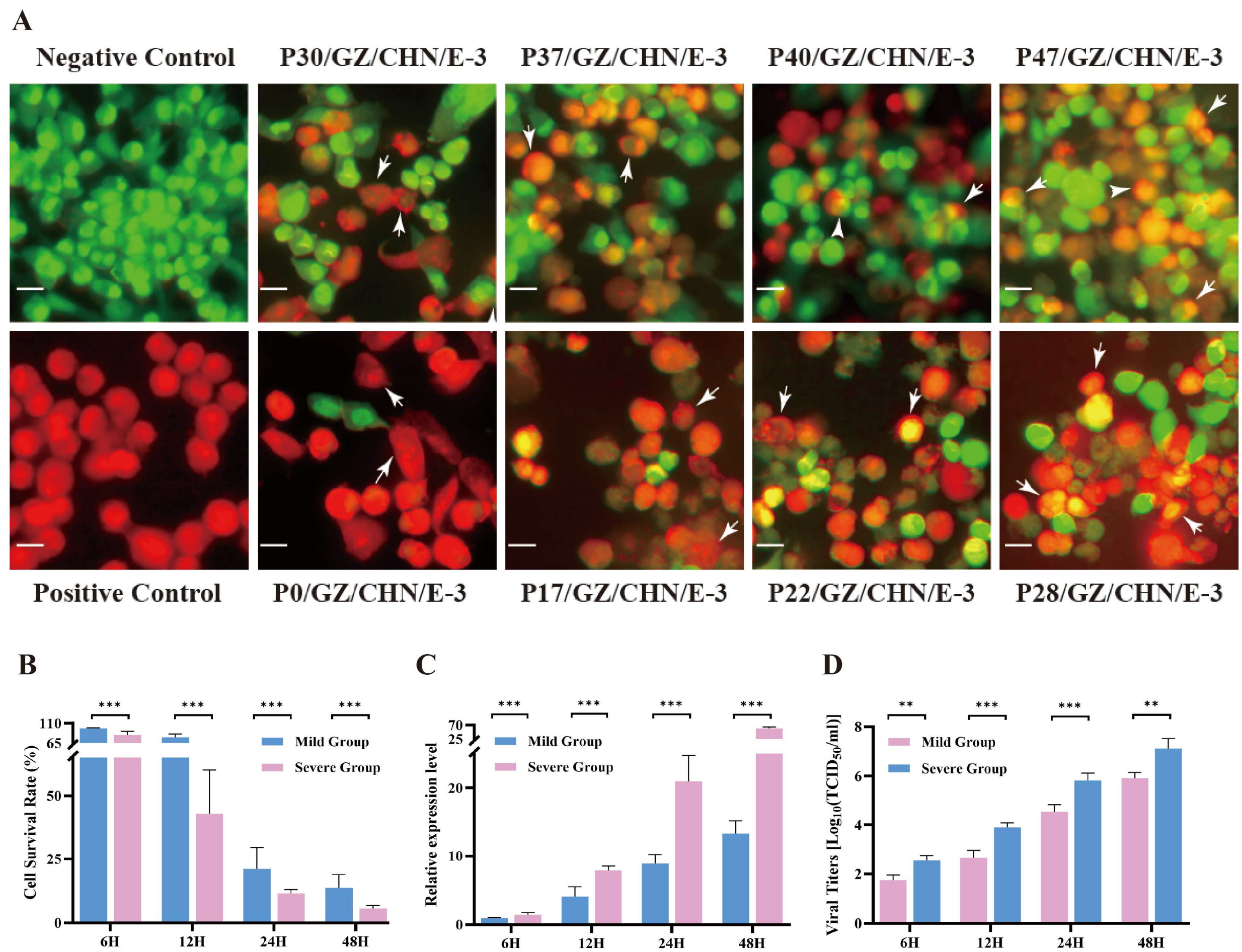

Fig. 1 RD cells were infected with the E-3 strains isolated from both mild and severe clinical case groups (MOI = 1). (A) AO/EB staining of RD cells at 48 hours post-infection with E-3 strains from mild and severe clinical groups. The P30, P37, P40, and P47 strains were isolated from the mild case group, and the P0, P17, P22, and P28 strains from the severe case group. The nuclei of infected cells exhibited membrane blebbing, condensed chromatin, and fragmented nuclei (indicated by white arrow). Bar=10 μm. AO penetrates all cells and stains nuclei green, while EB selectively enters cells with compromised membrane integrity, causing red or orange nuclear fluorescence. Consequently, live cells show uniformly green nuclei; early apoptotic cells displayed nuclear condensation or fragmentation with yellowish-green to yellow fluorescence; late apoptotic as well as necrotic cells exhibited red fluorescence. (B) Cell viability of E-3 strain-infected cells from mild and severe groups was assessed by CCK-8; (C) Relative RNA expression of E-3 strains from mild and severe case groups was measured by RT-qPCR using GAPDH as the internal reference gene. (D) Viral titers of the E-3 strains isolated from the mild and severe case groups in RD cells incubated at 37 °C were determined using the $TCID_{50}$ assay; The data on viral titers, cell survival rate, and relative expression level of virus RNA are presented as mean ± SD for the mild and severe case groups (**$p < 0.01$, ***$p < 0.001$).

Table 1. The cell viability of cells infected with the E-3 strain was compared between mild and severe case groups using the CCK-8 assay at multiple time points.

Time point (h)	Mild case group (n =12)	Severe case group (n =12)	*p* value
6 H	98.265 ± 1.295	83.413 ± 8.566	< 0.001
12 H	78.217 ± 7.571	43.021 ± 17.083	< 0.001
24 H	21.121 ± 8.488	11.405 ± 1.585	< 0.001
48 H	13.667 ± 5.200	5.645 ± 1.153	< 0.001

Data are presented as mean ± SD.

Table 2. The relative RNA expression levels of the E-3 strain were compared between the mild and severe case groups using RT-qPCR at multiple time points.

Time point (h)	Mild case group (n =12)	Severe case group (n =12)	*p* value
6 H	0.949 ± 0.098	1.440±0.314	< 0.001
12 H	4.072±1.422	7.873±0.635	< 0.001
24 H	8.865±1.362	20.948±3.769	< 0.001
48 H	13.291±1.835	59.428±5.196	< 0.001

Data are presented as mean ± SD.

Table 3. The viral titers of the E-3 strain were compared between mild and severe case groups using the $TCID_{50}$ assay at multiple time points.

Time point (h)	Mild case group (n =4)	Severe case group (n =4)	*p* value
6 H	1.75 ± 0.208	2.55 ± 0.208	0.002
12 H	2.68 ± 0.299	3.90 ± 0.183	< 0.001
24 H	4.55 ± 0.289	5.83 ± 0.299	< 0.001
48 H	5.93 ± 0.222	7.13 ± 0.403	0.002

Data are presented as mean ± SD.

In addition to AO/EB staining, we initially attempted to assess apoptosis using the calcein-acetoxymethyl ester (Calcein-AM, 2 μM) and propidium iodide (PI, 6 μM) double fluorescence staining assay (Fig. 2). However, the results were suboptimal for the experimental system. Specifically, we observed that viable cells exhibited only faint green fluorescence (Calcein), which was difficult to distinguish from background, even after systematically testing higher Calcein-AM concentrations (up to 6 μM) and longer incubation times (up to 30–45 minutes) (Fig. 2A–C, white

arrows). We attribute this primarily to the known limitations of the Calcein-AM/PI assay, which is relatively insensitive to early apoptotic cells and whose signal (Calcein) is highly dependent on cellular esterase activity. Calcein-AM enters live cells with intact membranes and is hydrolyzed by intracellular esterases into fluorescent calcein, which emits green fluorescence. In contrast, PI cannot cross intact membranes and only enters cells with compromised membrane integrity—such as those in late apoptosis, necrosis, or after fixation—where it binds to nucleic acids and fluoresces red. Given the need for a method with consistent and clear contrast to reliably quantify apoptotic cells in our model, we ultimately selected the AO/EB staining method for its better contrast and reproducibility.

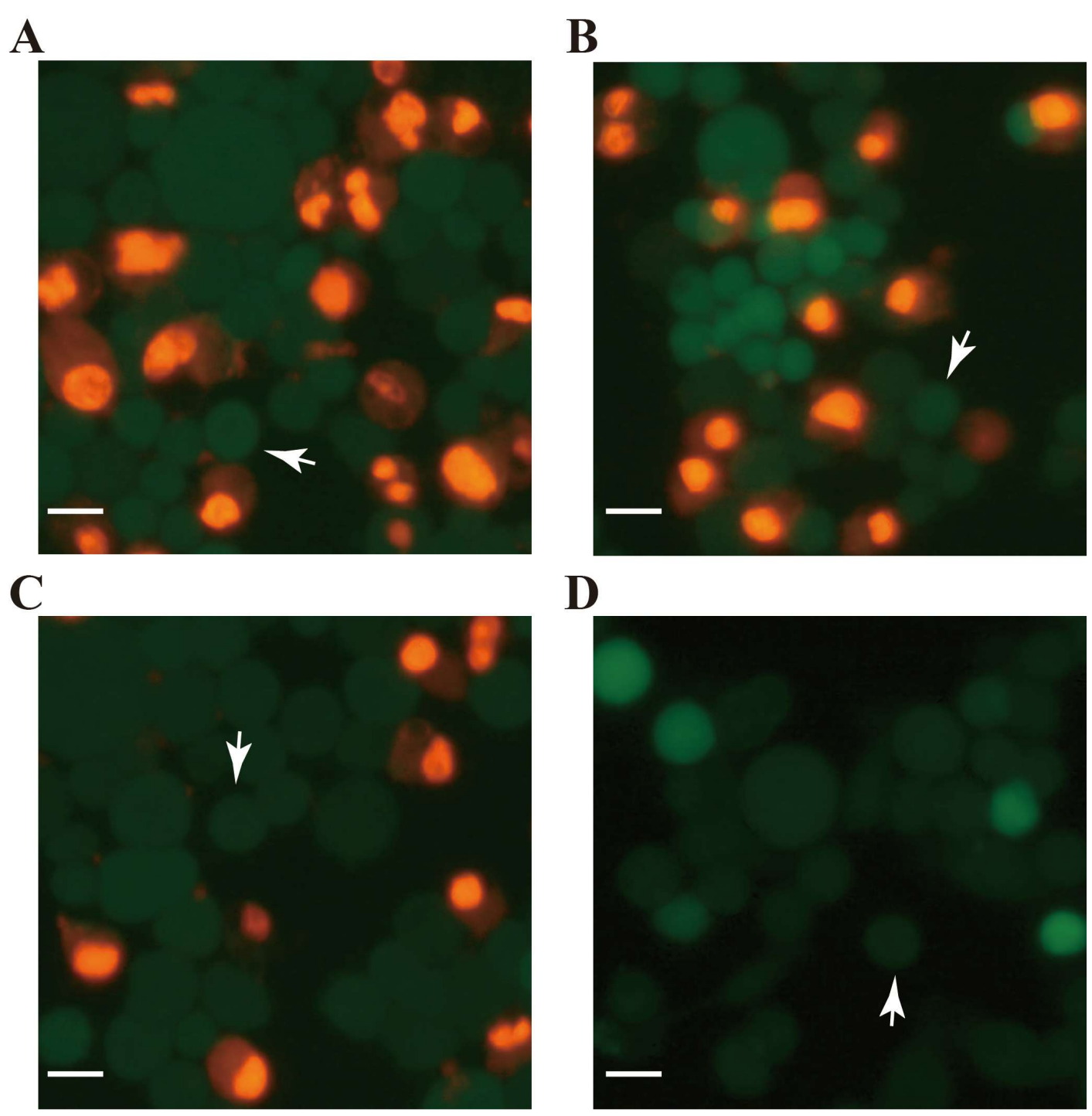

Fig. 2 Calcein Acetoxymethyl Ester (Calcein-AM) / Propidium Iodide (PI) staining of RD cells infected with E-3 strains.Bar=10 μm. (A, B, C) Calcein-AM/PI staining of RD cells infected with E-3 strains. (D) Calcein-AM/PI staining of normal RD cells. Live cells did not exhibit bright green fluorescence, and the green signal was weak and indistinct from the background (indicated by white arrows).

In summary, the isolates from the severe case group exhibit enhanced replication ability, more pronounced cytopathic effects, and increased genomic replication capacity in the cellular model, providing preliminary support for the potential

functional relevance of the observed genetic variations, though these findings are not sufficient to constitute a final conclusion. The in vitro experimental data and genetic analysis results are mutually supportive, establishing a foundation for future functional investigations and enabling the prioritization of specific variant sites and recombination patterns for in-depth research. It should be emphasized that this study is a correlation analysis and preliminary screening research, with its primary contribution residing in the systematic summarization of the genetic features of E-3 in severe and mild case groups and in the establishment of an initial association between genetic variants and in vitro functional phenotypes, thereby providing a clearly defined research direction for future investigations into the core virological determinants underlying the pathogenic mechanism of E-3. In addition, we have carefully ensured objective presentation in our summary, results, and discussion by avoiding absolute simplified terms such as "highly virulent/attenuated strain" or "more/less virulent." It is emphasized that clinical disease severity arises from complex interactions between viral and host factors (e.g., age, immune status), and while enhanced in vitro replication capacity may represent one potential virological basis for severe clinical symptoms, it is by no means the sole determining factor.

Best wishes.

Shijun Li, PhD
E-mail: zjumedjun@163.com
Jiafu Wang, PhD, professor
E-mail: jfwang@gzu.edu.cn
January 18, 2026

Re: Spectrum03136-25R1 (Genetic Variation, Recombinant Characteristics, and Seroprevalence Analysis of Echovirus 3 Causing Severe and Mild Cases of Hand, Foot, and Mouth Disease in Guizhou Province)

Dear Dr. Shijun Li:

Thank you for the privilege of reviewing your work. Below you will find my comments, instructions from the Spectrum editorial office, and the reviewer comments. It is essential to address the cocnerns of Reviewer #2.

Please return the manuscript within 60 days; if you cannot complete the modification within this time period, please contact me. If you do not wish to modify the manuscript and prefer to submit it to another journal, notify me immediately so that the manuscript may be formally withdrawn from consideration by Spectrum.

Revision Guidelines
To submit your modified manuscript, log into the submission site at https://spectrum.msubmit.net/cgi-bin/main.plex. Go to Author Tasks and click the appropriate manuscript title to begin. The information you entered when you first submitted the paper will be displayed; update this as necessary. Note the following requirements:

• Upload point-by-point responses to the issues raised by the reviewers in a file named "Response to Reviewers," NOT in your cover letter.
• Upload a compare copy of the manuscript (without figures) as a "Marked-Up Manuscript" file.
• Upload a clean .DOC/.DOCX version of the revised manuscript and remove the previous version.
• Each figure must be uploaded as a separate, editable, high-resolution file (TIFF or EPS preferred), and any multipanel figures must be assembled into one file.
• Any supplemental material intended for posting by ASM should be uploaded with their legends separate from the main manuscript. You can combine all supplemental material into one file (preferred) or split it into a maximum of 10 files with all associated legends included.

For complete guidelines on revision requirements, see our Submission and Review Process webpage. Submission of a paper that does not conform to guidelines may delay acceptance of your manuscript.

Data availability: ASM policy requires that data be available to the public upon online posting of the article, so please verify all links to sequence records, if present, and make sure that each number retrieves the full record of the data. If a new accession number is not linked or a link is broken, provide Spectrum production staff with the correct URL for the record. If the accession numbers for new data are not publicly accessible before the expected online posting of the article, publication may be delayed; please contact production staff (Spectrum@asmusa.org) immediately with the expected release date.

Publication Fees: For information on publication fees and which article types are subject to charges, visit our website. If your manuscript is accepted for publication and any fees apply, you will be contacted separately about payment during the production process; please follow the instructions in that e-mail. Arrangements for payment must be made before your article is published.

ASM Membership: Corresponding authors may join or renew ASM membership to obtain discounts on publication fees. Need to upgrade your membership level? Please contact Customer Service at Service@asmusa.org.

The ASM Journals program strives for constant improvement in our submission and publication process. Please tell us how we can improve your experience by taking this quick Author Survey.

Thank you for submitting your paper to Spectrum.

Sincerely,
Clinton Jones
Editor
Microbiology Spectrum

Reviewer #2 (Comments for the Author):

No

Reviewer #3 (Comments for the Author):

The authors provide appropriate responses to the comments made by both reviewers. However, I have additional comments, some of which are significant.

All the statistical analyses (including the different tests) performed in a study should usually be indicated in a specific paragraph at the end of the Materials and Methods section. Please, revise accordingly.

Page 3, line 110. The authors sequenced the complet viral genomes but did not indicate wether the nucleotide sequences were deposited in a database. Please, specifiy.

Page 4, line 132. The authors used the Datamonkey pipeline to perform investigations on selection mechanisms. The data are not provided in the manuscript nor the supplementray material. It would be helpful to the reader to have these data. Similarly, the RDP results should be provided to the reader.

Page 5, lines 188 - 190. Which statistical tests were performed?

Page 6, lines 227 & 228. In response to the main comment from the reviewer 2, with which I fully agree, the authors should change the designation of strains as severe or mild. Please, check the manuscript throughout and the comments below.

Figure 2. Through a quick search of GenBank, I found 59 complete or nearly complete echovirus 3 genomes. Did the authors perform a phylogenetic comparison with these genomes, which could provide an insightful epidemiological perspective on the sequences they describe?
Caption of Figure 2. Please, change "mild strains" and "severe strains".

Page 8, line 275. Please, change "mild strains" and "severe strains".

Page 8, lines 279 - 286. The statistics performed in the section are unclear to me. Please, explain.

Page 9, lines 287 - 294. I cannot figure out how the authors summarised the data they obtained from the selection analyses. They should provide the Datamonkey results in a supplementary material. In the caption, they write "The black dots represent amino acid sites with no positive selection pressure ($P > 0.1$)." Notwithstanding the fact that the number of these sites should be higher than shown in the figure, what is the relevance to indicate the amino acid sites that are not under positive selection? The authors also indicate in the caption "Lines and
circles represent amino acid variation (diversity) and evolutionary driving force (adaptability), respectively." How the authors quantify "evolutionary driving force (adaptability)"?

Figure 2. The biological significance of the 3D representation of proteins P1, P2, and P3 is completely beyond me. Please, explain.

Page 13, caption of figure 6. Please, indicate which statistical tests were performed.

Page 13, line 351. The authors should explain the following statement "which is consistent with the seroprevalence of EV-A71 and CV-A6 in the Chinese population". There is no correlation between an epidemic caused by one type of enterovirus and an epidemic caused by another type.

Figure 7. Please, specify the number of replicates for each test and time point.

Page 14, line 370. I disagree with the suggestion that there has been a gradual change in the epidemiology of EV-A71 and CV-A16, when other factors are also likely to be involved. In particular, there have been changes in the reporting and diagnosis (genotyping) of enterovirus infections associated with cases of hand, foot and mouth disease.

Page 15, line 376 and followings. "E-3 has been relatively uncommon in HFMD patients" To assert this, the authors should specify since when enteroviruses of species B have actually been investigated as causative agents of hand, foot and mouth disease. I do not understand the link between the two points in this phrase: "while its nearly full-length genome was first reported in Yunnan in 2022 [51], no severe E-3-associated HFMD cases have been documented globally".

Page 16, line 450. Please, check and revise "mild and severe strains".

Page 16, line 451. About the statement "independent evolutionary pathways": the phylogenies show clearly that strains from mild and severe cases are "only" diverging from one another while they derive from a single evolutionary pathway.

Line 462. About the statement "This study elucidates the relationship between genomic variation and pathogenicity", I would suggest something like "This study provides avenues for further investigation into the relationship between observed genetic

variations and the pathogenicity of E-3 strains."

Responses to reviewer's comments

Dear Editors and Reviewers:

Thank you for your letter and the comments concerning our manuscript entitled "Genetic Variation, Recombinant Characteristics, and Seroprevalence Analysis of Echovirus 3 Causing Severe and Mild Cases of Hand, Foot, and Mouth Disease in Guizhou Province" (Manuscript Number: Spectrum03136-25R2). Those comments are all valuable and very helpful for revising and improving our paper and also hold important guiding significance for our research.

Responses to Reviewer #3:

1. Comment

All the statistical analyses (including the different tests) performed in a study should usually be indicated in a specific paragraph at the end of the Materials and Methods section. Please, revise accordingly.

Answer:

We have supplemented the detailed descriptions of all statistical analyses at the end of the Methods section (highlighted in blue; lines 220-242, page 6).

The description of the statistical analyses in the Methods section is as follows:

Statistical analysis

Selection pressure was estimated using the MEME method implemented on the Datamonkey web server. For each site, MEME estimates one synonymous substitution rate (α) and two distinct nonsynonymous rates (β^- and β^+). A likelihood ratio test (LRT) was conducted between a null model constraining positive selection and an alternative model allowing positive selection. Sites with $\beta^+ > \alpha$ and a statistically significant LRT *p*-value were identified as experiencing significant episodic positive selection.

Nucleotide substitution sites in the ORF region were identified by aligning viral sequences from 4 strains from the severe case group and 4 strains from the mild case group against the E-3 prototype strain using ClustalW in MEGA (version 11.0). For each identified substitution site, a 2×2 contingency table was constructed to compare the distribution of variant nucleotides between the severe and mild case groups. Fisher's exact test was used to assess differences in nucleotide substitution site distribution between the severe and mild case groups (n=4 per group). Odds ratios (ORs) and two-sided p-values were calculated for each site, with statistical significance defined as $p < 0.05$. For nucleotide sites significantly associated with disease severity ($p < 0.05$), the direction of association was defined as the disease group (severe or mild disease group) in which the variant nucleotide (relative to the prototype strain) exhibited a within-group dominant frequency ≥ 80%. Variants meeting this criterion were classified as associated with severe or mild disease, respectively.

Differences in cell survival rates, gene expression levels, and virus titers between the severe and mild case groups were analyzed using two-tailed independent-samples t-tests at each time point (6 h, 12 h, 24 h, and 48 h) with GraphPad Prism (version 10.6.1). Each group comprised four independent viral strains, with three technical replicates per strain. Data are expressed as mean ± standard deviation (SD). Exact p-values were calculated accordingly, and statistical significance was defined as $p < 0.05$, with ** indicating $p < 0.01$ and *** indicating $p < 0.001$.

2. Comment

Page 3, line 110. The authors sequenced the complet viral genomes but did not indicate wether the nucleotide sequences were deposited in a database. Please, specifiy.

Answer:

We have now added the GenBank accession numbers in the Methods section (highlighted in blue; lines 130-131, page 4), as follows.

The genome sequences detected in this study have been deposited in NCBI GenBank under accession numbers PV137766-PV137773.

This information was also provided in the "Data availability" section (lines 546-547, page 19).

3. Comment

Page 4, line 132. The authors used the Datamonkey pipeline to perform investigations on selection mechanisms. The data are not provided in the manuscript nor the supplementray material. It would be helpful to the reader to have these data. Similarly, the RDP results should be provided to the reader.

Answer:

The analysis data generated by the RDP5 software have already been included in Supplementary Table S3 of the original manuscript. Following the reviewer's suggestion, we have now added the Datamonkey results to Supplementary Table S6.

4. Comment

Page 5, lines 188-190. Which statistical tests were performed?

Answer:

Detailed statistical methods have been added to the Methods section (highlighted in blue; lines 237-242, page 6).

All results are expressed as mean ± standard deviation (SD). Two-tailed independent-samples t-tests were used for pairwise comparison between the severe and mild case groups at the same time point, with exact p values calculated using GraphPad Prism (version 10.6.1). Statistical significance was defined as $p < 0.05$, with ** indicating $p < 0.01$ and *** indicating $p < 0.001$.

The relevant statement in the Methods section is as follows:

Differences in cell survival rates, gene expression levels, and virus titers between the severe and mild case groups were analyzed using two-tailed independent-samples t-tests at each time point (6 h, 12 h, 24 h, and 48 h) with GraphPad Prism (version

10.6.1). Each group comprised four independent viral strains, with three technical replicates per strain. Data are expressed as mean ± standard deviation (SD). Exact p values were calculated accordingly, and statistical significance was defined as $p < 0.05$, with ** indicating $p < 0.01$ and *** indicating $p < 0.001$.

5. Comment

Page 6, lines 227 & 228. In response to the main comment from the reviewer 2, with which I fully agree, the authors should change the designation of strains as severe or mild. Please, check the manuscript throughout and the comments below.

Answer:

We have uniformly revised the strain designation throughout the entire manuscript (highlighted in blue; lines 253-256, page 6; lines 333-335, page 10; line 440, page 17; lines 460-463, page 17; lines 470-473, page 17).

The relevant statement in the manuscript is as follows:

Based on the principle of > 85% VP1 sequence identity for genotype classification [51], all E-3 strains were identified as subgenotype C6 (Fig. 1), with four strains from the severe case group and four strains from the mild case group forming two separate sub-branches diverging from a common lineage.

Interestingly, The shared mutation sites between the mild and severe case groups were observed in the P2 and P3 regions, including sites 2A-952, 2C-1180, 2C-1217, 2C-1389, 3A-1451, and 3D-2079 (Fig. 4B-4C, Table S5).

The potential virulence change in E-3 variants from severe cases warrants attention.

Previous studies have confirmed differences in replication and virulence of enteroviruses isolated from severe and mild cases, potentially linked to pathogenesis [86-87], with these candidate sites associated with severe disease identified in the 2A, 2C, 3A, and 3D regions [47, 57-58, 88-90].

This study identified a shared mutation at position 3A-1451 (alanine in the E3 prototype strain and in E3 strains from the mild case group, changed to threonine in strains from the severe case group), resulting in a shift from nonpolar hydrophobic to polar hydrophilic residues that may alter the dimeric protein's structure and function.

6. Comment

Figure 2. Through a quick search of GenBank, I found 59 complete or nearly complete echovirus 3 genomes. Did the authors perform a phylogenetic comparison with these genomes, which could provide an insightful epidemiological perspective on the sequences they describe?

Answer:

This has been clarified in the Results section of the manuscript (highlighted in blue; lines 273-283, page 9) and shown in Supplementary Fig. S1.

We obtained 61 complete or near-complete E-3 genomic sequences from GenBank (including the 8 E-3 strains in this study and 53 global E-3 reference strains) and constructed phylogenetic trees based on the VP1, P1, P2, and P3 coding regions, respectively. To avoid redundancy in the main text-figures, these four phylogenetic trees are included in Supplementary Fig. S1.

The relevant statement in the Results section of the manuscript is as follows:

To further clarify the evolutionary characteristics of the E-3 strains, 61 complete or nearly complete E-3 genomic sequences (including the 8 Guizhou strains and 53 global reference strains) were downloaded from GenBank and used to construct phylogenetic trees (Fig. S1). All eight Guizhou E-3 strains clustered within genotype C6 in the VP1 region (Fig. S1A), and strains from the mild and severe case groups formed two separate sub-branches diverging from a common lineage. The P1 structural region was relatively conserved, with a branching pattern consistent with the VP1 tree (Fig. S1B). In contrast, the P2 and P3 regions showed greater genetic diversity and divergent topological structures. In the P2 region, E-3 strains from the severe case group clustered with a 2019 Thai strain, while strains from the mild case

group clustered with 2022 Yunnan strains (Fig. S1C). In the P3 region, although the overall clustering of strains was similar to the VP1 tree, internal topology and bootstrap support differed markedly from the VP1 and P1 regions (Fig. S1D).

The Supplementary Fig. S1 is as follows.

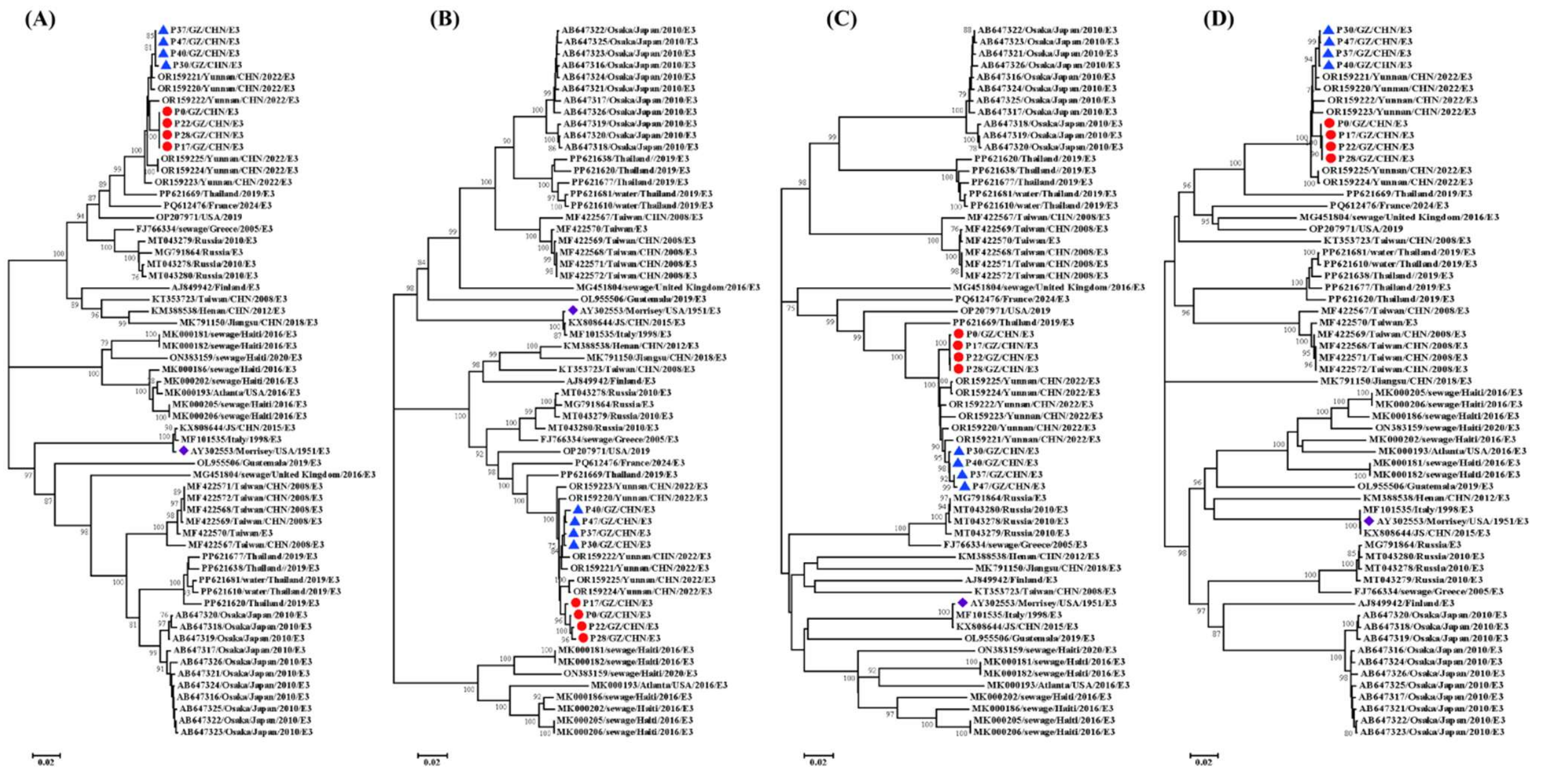

Fig. S1 The phylogenetic trees are based on the VP1, P1, P2, and P3 sequences of E-3. The four strains from severe cases are indicated by red solid circles, the four strains from mild cases are indicated by blue triangles, and the E-3 prototype is indicated by a brown diamond. Eight Guizhou E-3 strains and other E-3 strains were analyzed through nucleotide alignment using the neighbor-joining method and the Kimura 2-parameter model implemented in MEGA 11.0. Bootstrap values >75 are shown and inferred from bootstrapping with 1,000 replicates. (A) VP1 coding sequences; (B) P1 coding sequences; (C) P2 coding sequences; (D) P3 coding sequences.

7. Comment

Caption of Figure 2. Please, change "mild strains" and "severe strains".

Answer:

The relevant description has been revised in the manuscript (highlighted in blue; line 257, page 8).

The relevant statement is as follows:

Fig. 2 The phylogenetic trees based on the VP1, P1, P2, and P3 sequences of EV-B. The four strains from severe cases were indicated by red solid circles, the four

strains from mild cases were indicated by blue triangles, and the E-3 prototype was indicated by a brown diamond.

8. Comment

Page 8, line 275. Please, change "mild strains" and "severe strains".

Answer:

The relevant description has been revised in the manuscript (highlighted in blue; lines 314-316, pages 9-10).

The relevant statement in the manuscript is as follows:

Furthermore, E-3 strains from the severe and mild case groups formed two separate sub-branches in the VP1, P1, P2, and P3 phylogenetic trees, suggesting that the virus might have accumulated specific mutations during its evolution.

9. Comment

Page 8, lines 279-286. The statistics performed in the section are unclear to me. Please, explain.

Answer:

The statistical analysis has been added to the Methods section of the manuscript (highlighted in blue; lines 226-236, page 6).

The specific methods for identifying nucleotide substitution sites in the ORF region of E-3 strains are as follows.

Nucleotide substitution sites in the ORF region were identified by aligning viral sequences from the four strains in the severe case group and the four strains in the mild case group against the E-3 prototype strain using ClustalW as implemented in MEGA (version 11.0). For each substitution site, a 2×2 contingency table was constructed to compare variant nucleotide distribution between the severe and mild groups, with rows representing disease severity groups and columns indicating presence or absence of the variant nucleotide at that site. Fisher's exact test was used to assess the statistical significance of differences in the distribution of nucleotide

substitution sites between severe and mild groups, given the small sample size (n = 4 per group). Odds ratios (ORs) and two-sided *p*-values were calculated for each site, with $p < 0.05$ defined as statistically significant.

For each nucleotide site showing a statistically significant association with disease severity ($p < 0.05$), the associated direction was defined as the disease group (severe group or mild group) in which the variant nucleotide (relative to the E-3 prototype strain) at a within-group dominant frequency ≥ 80%. Specifically, variants statistically significant in the severe group and meeting the ≥ 80% within-group dominant frequency threshold were classified as associated with severe disease, while those significantly more frequent in the mild group and satisfying the same frequency criterion were defined as associated with mild disease.

The revised text in the Methods section is as follows:

Nucleotide substitution sites in the ORF region were identified by aligning viral sequences from 4 strains from the severe case group and 4 strains from the mild case group against the E-3 prototype strain using ClustalW in MEGA (version 11.0). For each identified substitution site, a 2×2 contingency table was constructed to compare the distribution of variant nucleotides between the severe and mild case groups. Fisher's exact test was used to assess differences in nucleotide substitution site distribution between the severe and mild case groups (n = 4 per group). Odds ratios (ORs) and two-sided *p*-values were calculated for each site, with statistical significance set at $p < 0.05$. For nucleotide sites significantly associated with disease severity ($p < 0.05$), the direction of association was defined as the disease group (severe or mild disease group) in which the variant nucleotide (relative to the prototype strain) exhibited a within-group dominant frequency ≥ 80%. Variants meeting this criterion were classified as associated with the severe or mild disease, respectively.

10. Comment

Page 9, lines 287-294. I cannot figure out how the authors summarised the data they obtained from the selection analyses. They should provide the Datamonkey

results in a supplementary material. In the caption, they write "The black dots represent amino acid sites with no positive selection pressure (P > 0.1)." Notwithstanding the fact that the number of these sites should be higher than shown in the figure, what is the relevance to indicate the amino acid sites that are not under positive selection? The authors also indicate in the caption "Lines and circles represent amino acid variation (diversity) and evolutionary driving force (adaptability), respectively." How the authors quantify "evolutionary driving force (adaptability)"?

Answer:

This issue has been addressed in the revised manuscript (highlighted in blue; lines 221-225, page 6; line 329, page 10; line 343, page 11).

We have added Supplementary Table S6 in the revised manuscript. This table presents the results from the MEME (Mixed Effects Model of Evolution) analysis performed on the Datamonkey online server, which identifies sites under positive selection ($p < 0.1$). Additionally, the description has been updated (highlighted in blue; lines 221-225, page 6) in the Methods section of the manuscript and directly references Table S6 in the Results section (highlighted in blue; line 329, page 10).

The revised description in the Methods section of the manuscript is as follows (highlighted in blue; lines 221-225, page 6):

Selection pressure was estimated using the MEME method implemented on the Datamonkey web server. For each site, MEME estimates one synonymous substitution rate (α) and two distinct nonsynonymous rates (β^- and β^+). A likelihood ratio test (LRT) was conducted between a null model constraining positive selection and an alternative model allowing positive selection. Sites with $\beta^+ > \alpha$ and a statistically significant LRT p-value were identified as experiencing significant episodic positive selection.

The x-axes in Fig. 3 (P1, P2, and P3 regions) represent complete, continuous amino acid positions without omission. The sparse appearance of black dots in the

original figure was due to overlap and occlusion by entropy lines, other markers, and colored boxes, rather than incomplete plotting.

Following the reviewer's suggestion, we have revised Fig. 3 and its legend (highlighted in blue; line 343, page 11).

Only positively selected sites (green boxes, $p < 0.1$) and highly variable sites with high entropy (red boxes, entropy > 0.6) are highlighted to emphasize the key evolutionary findings. The original black dots (previously labeled as non-positively selected sites) have been replaced with light gray, semi-transparent background dots, which are only used as a background to display the other amino acid sites and no longer explain the sites without positive selection. The figure legend has been revised accordingly, removing redundant descriptions of non-selected sites, so that the figure focuses solely on the critical evolutionary characteristics of E-3 strains, avoiding potential confusion.

The ambiguous phrase "variation (diversity) and evolutionary driving force (adaptability)" has been corrected and replaced with precise, quantifiable terminology in the revised legend.

The specific figure and its caption are shown below.

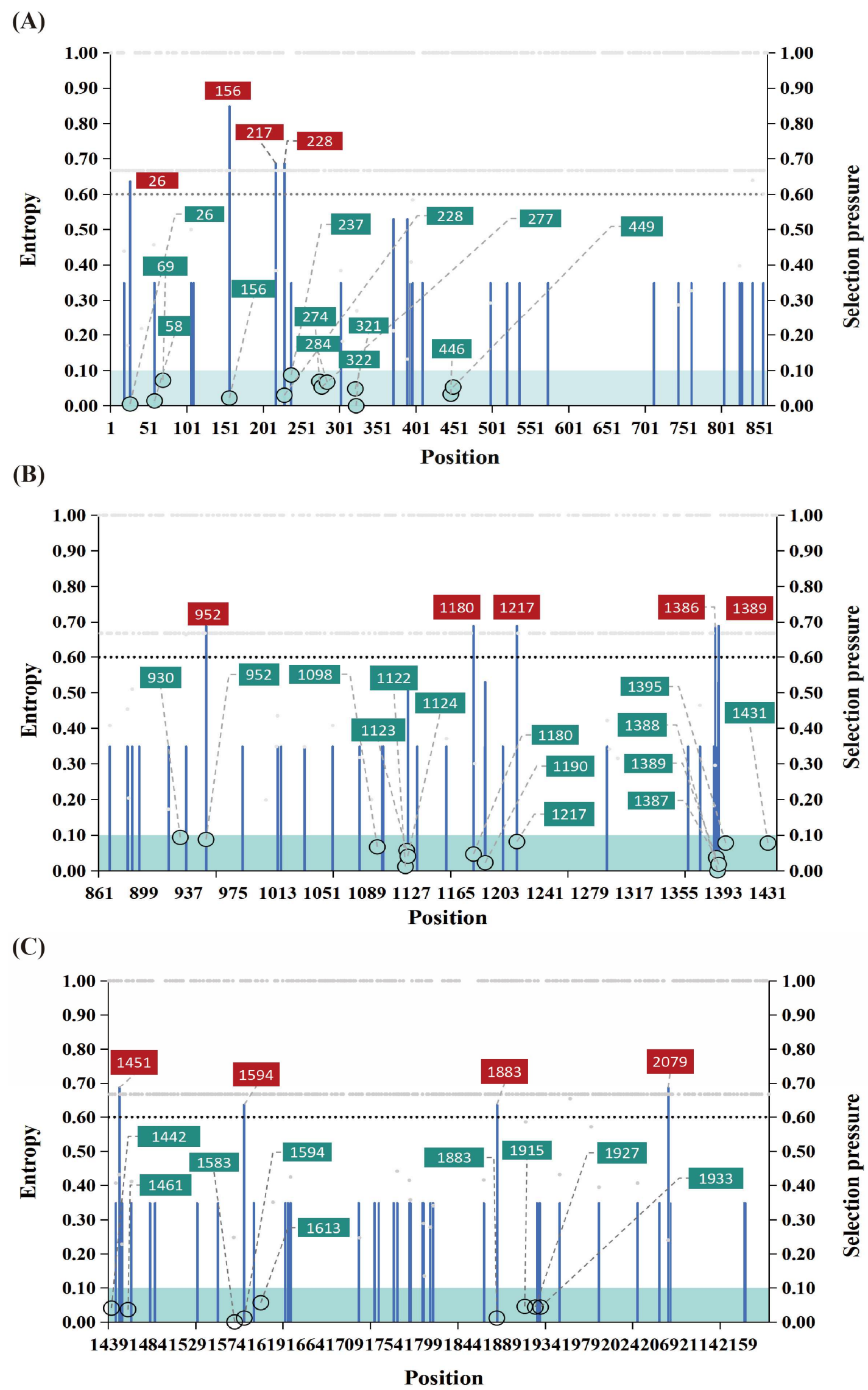

Fig. 3 Analysis of amino acid sites under positive selection pressure identified by MEME analysis and sites with high variation identified by entropy value analysis in E-3 strains isolated from mild and severe cases. Green boxes indicate sites under significant positive selection (MEME test, $p < 0.1$). Red boxes highlight sites with high entropy (Shannon entropy > 0.6). Gray dots indicate the remaining sites (shown as background reference). Line height represents the Shannon entropy value (a measure of amino acid diversity). Circles indicate sites under positive selection pressure ($p < 0.1$). All sites are displayed consecutively along the x-axis. (A) P1 region. (B) P2 region. (C) P3 region.

11. Comment

Figure 2. The biological significance of the 3D representation of proteins P1, P2, and P3 is completely beyond me. Please, explain.

Answer:

We have clarified the biological significance of the 3D structural models in the Results section and revised the figure legend (highlighted in blue; lines 327-342, page 10; line 345, page 12).

We first clarify the figure numbering. The phylogenetic trees based on the VP1, P1, P2, and P3 sequences are shown in Fig. 2, while the 3D structures of the P1, P2, and P3 proteins are presented in Fig. 4.

Positively selected sites (MEME test, $p < 0.1$), highly variable sites (Shannon entropy > 0.6), and shared amino acid mutations between the mild and severe groups were mapped onto the 3D structural model (Fig. 4). The results highlight potential associations between amino acid variation and the structural and functional features of E-3 strains, providing structural insights into the differing pathogenic potential observed between groups.

The P1 region encodes the capsid proteins VP1-VP4, which are key regions for receptor binding, invasion, and immune evasion. The positively selected and highly variable sites such as VP4-26, VP2-156, and VP2-228 are exposed on the capsid surface and may affect viral antigenicity and cell tropism. The P2 region encodes non-structural proteins such as 2A, 2B, and 2C, which are involved in viral replication and host regulation. The shared mutation sites (e.g., 2A-952, 2C-1180, 2C-1217, and 2C-1389) between the mild and severe case groups are located in the functional core region, are all under positive selection pressure, and exhibit high variability. These mutation sites may enhance virulence by altering replication efficiency. The P3 region encodes replication-related proteins such as 3A, 3C, and 3D. The shared mutation sites between the mild and severe case groups (e.g., 3A-1451 and 3D-2079) also exhibit high variability. Furthermore, the positively selected and highly variable sites (e.g., 3C-1594 and 3D-1883) may affect viral replication and assembly, and thus potentially contribute to the clinical severity of infection.

Combined structural, phylogenetic, and selection pressure analyses indicate that mutations in the P2 and P3 regions are not randomly distributed, but enriched in

functionally critical domains and associated with clinical severity of infection. Previous studies have indicated that mutations in P2 and P3 can contribute to variations in disease severity [1-5]. Notably, P2 and P3 are also recombination hotspots in enteroviruses. The synergistic effect of mutations and recombination may further influence viral evolution and pathogenicity.

We have revised the legend of Figure 4 to more clearly label the spatial positions and potential functional roles of key sites. The specific figure and its caption are shown below (highlighted in blue; line 345, page 12).

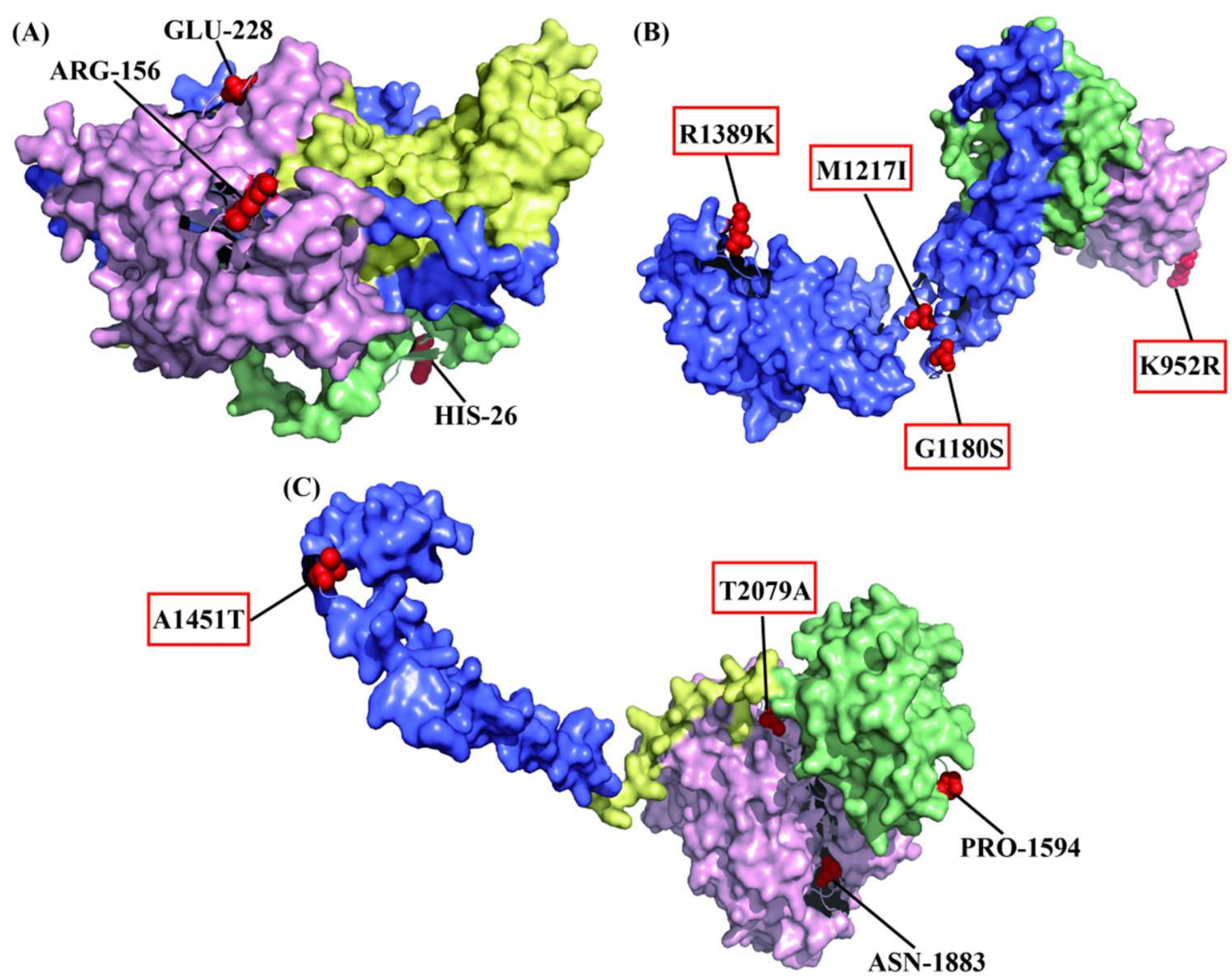

Fig. 4 3D structural mapping of key amino acid variations in E-3 virus P1, P2, and P3 polyproteins. (A) 3D structure of the P1 capsid polyprotein. VP1 (yellow), VP2 (pink), VP3 (blue), and VP4 (green). (B) 3D structure of the P2 non-structural polyprotein. 2A (pink), 2B (green), and 2C (blue). (C) 3D structure of the P3 non-structural polyprotein. 3A (blue), 3B (yellow), 3C (green), and 3D (pink). Amino acid sites VP4-26, VP2-156, VP2-228, 2A-952, 2C-1180, 2C-1217, 2C-1389, 3C-1594, and 3D-1883 were under positive selection pressure (MEME test, $p < 0.1$) and highly variable (Shannon entropy > 0.6), and sites 3A-1451 and 3D-2079 were highly variable sites (Shannon entropy > 0.6). Red boxes indicate shared amino acid substitutions between the severe and mild case groups, as well as high-variation or positively selected sites.

The statement in the Results section of the manuscript is as follows (highlighted in blue; lines 327-342, page 10):

Selection pressure and Shannon entropy analysis revealed that several amino acid sites were under positive selection pressure and highly variable (entropy value > 0.6), including sites VP4-26, VP2-156, VP2-228, 2A-952, 2C-1180, 2C-1217, 2C-1389, 3C-1594, and 3D-1883 (Fig. 3-4, Table S5-S6). Three-dimensional structural mapping showed that these sites were predominantly located on the surface of capsid proteins (such as VP4-26, VP2-156, VP2-228, Fig. 4A) or within functional domains of non-structural proteins (such as the 2C helicase motif, the 3D polymerase active site, Fig. 4B-4C). Interestingly, The shared mutation sites between the mild and severe case groups were observed in the P2 and P3 regions, including sites 2A-952, 2C-1180, 2C-1217, 2C-1389, 3A-1451, and 3D-2079 (Fig. 4B-4C, Table S5). Among these, sites 3A-1451 and 3D-2079 were also highly variable (entropy > 0.6). Eight shared nucleotide substitutions were commonly observed in the untranslated regions (UTRs) of E-3 strains from mild and severe case groups (Table S5). Mutations in the 5' UTR region can impact replication efficiency, cell tropism, and virulence, and specific potential virulence sites have been identified [52-55]. Previous studies have suggested that mutations in the P2 and P3 regions can lead to variations in disease severity [52, 56-59]. Consistently, our structural localization further shows that these non-structural protein mutations are enriched in functional domains and may contribute to the clinical severity of E-3 infection.

References are as follows:

1. Li B, Yue Y, Zhang Y, Yuan Z, Li P, Song N, et al. A Novel Enterovirus 71 (EV71) Virulence Determinant: The 69th Residue of 3C Protease Modulates Pathogenicity. Front Cell Infect Microbiol **2017;**7:26.

2. Li C, Qiao Q, Hao SB, Dong Z, Zhao L, Ji J, et al. Nonstructural protein 2A modulates replication and virulence of enterovirus 71. Virus Res. **2018;**244:262-9.

3. Jia QJ, Chen XY, Li Z, Xu JJ, Xu ZG, Duan ZL, et al. Comparative Genomic Analysis of Enterovirus 71 Revealed Six New Potential Neurovirulence-associated Sites. Biomed Environ Sci **2016;**29:767-772.

4. Ma HY, Lu CY, Tsao KC, Shih HM, Cheng AL, Huang LM, et al. Association of EV71 3C polymorphisms with clinical severity. J Microbiol Immunol Infect **2018;**51:608-613.

5. Lai YW, Cheng D, Huang SW, Chu JJH, Yeh MT, Chang CF, et al. Impact of Genomic

Variations Found in Enterovirus A71 Virus Population on Viral Properties and Disease Severity. J Med Virol **2026;**98:e70814.

12. Comment

Page 13, caption of figure 6. Please, indicate which statistical tests were performed.

Answer:

The statistical methods have been added to the legend of Fig. 6 (highlighted in blue; line 387, page 15).

The statistical methods are as follows:

Statistical analyses were performed to compare differences between groups at each time point. Each group comprised four independent viral strains, with three technical replicates per strain. All results are expressed as mean ± standard deviation (SD). Two-tailed independent-samples t-tests were conducted for each pairwise comparison between the severe and mild case groups at the same time point to calculate exact *p*-values, as implemented in GraphPad Prism (version 10.6.1). Statistical significance was defined as $p < 0.05$, with ** indicating $p < 0.01$ and *** indicating $p < 0.001$.

The relevant description in the Fig. 6 legend is as follows:

The data on viral titers, cell survival rate, and relative expression levels of viral RNA are presented as mean ± SD. Two-tailed independent-samples t-tests were used to compare the severe and mild case groups at each time point, and exact *p*-values were calculated (**$p < 0.01$, ***$p < 0.001$).

13. Comment

Page 13, line 351. The authors should explain the following statement "which is consistent with the seroprevalence of EV-A71 and CV-A6 in the Chinese population". There is no correlation between an epidemic caused by one type of enterovirus and an epidemic caused by another type.

Answer:

We have revised the original description to avoid any misunderstanding regarding causal or epidemiological linkages between serotypes (highlighted in blue; lines 395-398, page 15).

This statement refers only to numerical similarity in seroprevalence levels between E-3 and the previously reported enteroviruses (EV-A71 and CV-A6) in Chinese populations.

The revised text in the Results section are as follows:

The seroprevalence rate and GMT of E-3 were 54.55% and 1:28.78 in Tongren, and 50% and 1:27.71 in Zunyi, respectively. These findings suggest that small-scale epidemics of E-3 occurred in these areas, with seroprevalence rates numerically similar to those documented for other HFMD-associated enteroviruses (e.g., EV-A71 and CV-A6) in Chinese populations [62].

14. Comment

Figure 7. Please, specify the number of replicates for each test and time point.

Answer:

The relevant details have been added in the Methods section (highlighted in blue; lines 213-217, page 5).

For each of the eight Guizhou E-3 strains, viral titer assays were performed in three independent biological replicates (n = 3) at every time point (4, 8, 16, 24, 48, and 72 h post-infection) under incubation conditions of 36 °C and 39.5 °C. Mean viral titers and standard deviations (SD) were calculated from the triplicate measurements for each time point. The mean titers were used to plot the viral growth curves shown in Fig. 7.

The revised text in the Methods section is as follows:

All viral titer assays were performed in three independent biological replicates (n = 3) for each of the eight Guizhou E-3 strains at every time point (4, 8, 16, 24, 48, and 72 h post-infection) under incubation conditions of 36 °C and 39.5 °C. The mean viral

titer and SD were calculated from the triplicate measurements for each condition, and the mean titers were used to generate the viral growth curves shown in Fig. 7.

We have included a table that lists the detailed mean ± SD values for all strains, temperatures, and time points.

Table 1 Mean ± SD of viral titers (log_{10} $TCID_{50}$/mL) for eight Guizhou E-3 strains at 36 °C and 39.5 °C across all time points

Strain	Temperature	4 h	8 h	16 h	24 h	48 h	72 h
P0/GZ/CHN/E	36 °C	1.93±0.06	3.83±0.06	5.00±0.10	6.83±0.06	7.83±0.06	7.53±0.06
-3	39.5 °C	1.43±0.06	2.63±0.06	4.63±0.06	5.83±0.06	7.10±0.20	6.93±0.15
P17/GZ/CHN/	36 °C	2.83±0.06	3.30±0.10	4.73±0.06	6.53±0.06	7.50±0.20	7.30±0.20
E-3	39.5 °C	1.63±0.06	2.83±0.06	4.23±0.06	5.33±0.15	7.23±0.06	6.70±0.10
P22/GZ/CHN/	36 °C	1.73±0.06	3.53±0.06	4.33±0.06	6.40±0.10	7.70±0.20	7.13±0.06
E-3	39.5 °C	1.33±0.06	2.53±0.06	3.53±0.06	5.03±0.06	7.00±0.10	6.50±0.20
P28/GZ/CHN/	36 °C	1.93±0.06	3.33±0.06	4.63±0.06	6.20±0.20	7.40±0.10	7.00±0.10
E-3	39.5 °C	2.23±0.06	2.70±0.10	3.93±0.06	5.63±0.06	6.83±0.06	6.53±0.06
P30/GZ/CHN/	36 °C	1.27±0.06	3.00±0.10	3.70±0.10	5.07±0.06	6.77±0.06	6.40±0.20
E-3	39.5 °C	1.00±0.10	2.27±0.06	2.63±0.15	4.40±0.10	5.67±0.15	5.87±0.06
P37/GZ/CHN/	36 °C	1.57±0.06	3.27±0.06	3.63±0.15	5.50±0.20	6.57±0.06	6.20±0.10
E-3	39.5 °C	1.27±0.06	2.20±0.10	2.77±0.06	4.50±0.10	5.50±0.20	5.77±0.15
P40/GZ/CHN/	36 °C	1.50±0.10	3.20±0.20	3.77±0.06	5.80±0.20	6.70±0.10	6.27±0.06
E-3	39.5 °C	1.17±0.06	2.50±0.10	2.90±0.20	4.70±0.10	5.90±0.20	6.07±0.06
P47/GZ/CHN/	36 °C	1.87±0.06	3.20±0.10	3.70±0.10	5.17±0.06	6.30±0.20	5.97±0.06
E-3	39.5 °C	1.27±0.06	2.57±0.06	3.00±0.20	4.57±0.06	5.77±0.06	5.90±0.20

15. Comment

Page 14, line 370. I disagree with the suggestion that there has been a gradual change in the epidemiology of EV-A71 and CV-A16, when other factors are also likely to be involved. In particular, there have been changes in the reporting and diagnosis (genotyping) of enterovirus infections associated with cases of hand, foot and mouth disease.

Answer:

We have revised the relevant statement (highlighted in blue; lines 416-419, page 16) .

The shift in predominant enterovirus serotypes reflects multiple contributing factors, not merely a pure epidemiological change, including improvements in enterovirus genotyping and diagnostic reporting, as well as potential changes in viral circulation patterns.

The revised text in the Discussion section is as follows:

Since 2013, other enteroviruses have shown a gradual increase in detection frequency and have become the dominant serotypes reported in association with HFMD in China [66-67], a trend also documented in Guizhou Province [68]. Importantly, this apparent shift likely reflects both evolving viral epidemiology and improvements in enterovirus genotyping and diagnostic surveillance.

16. Comment

Page 15, line 376 and followings. "E-3 has been relatively uncommon in HFMD patients" To assert this, the authors should specify since when enteroviruses of species B have actually been investigated as causative agents of hand, foot and mouth disease. I do not understand the link between the two points in this phrase: "while its nearly full-length genome was first reported in Yunnan in 2022 [51], no severe E-3-associated HFMD cases have been documented globally".

Answer:

We have revised the relevant paragraph to improve clarity (highlighted in blue; lines 422-432, pages 16-17).

The apparently low prevalence of E-3 likely reflects under-surveillance rather than a truly low incidence, especially given the historical focus of HFMD surveillance.

HFMD was included in the list of legally notifiable infectious diseases in China in 2008. The national Hand, Foot, and Mouth Disease Prevention and Control Guidelines (2009 Edition), issued by the Chinese Center for Disease Control and Prevention, already recognized EV-B members (including coxsackievirus B) as potential etiologic agents.

(https://www.chinacdc.cn/jkyj/crb2/bl/szkb/jswj_szkb/202409/t20240907_299058.html). However, national surveillance efforts initially focused almost exclusively on EV-A71 and CV-A16, which were associated with severe and fatal cases. Early diagnostic protocols prioritized EV-A detection. Systematic monitoring of EV-B serotypes was not widely implemented until approximately 2010-2013 [1-3], and targeted diagnostic capacity for E-3 remains limited in many regions. Because E-3 has not been linked to severe HFMD outbreaks, it has received low priority in routine testing, leading to sustained underdetection.

The revised text in the Discussion section is as follows:

Nationally, the HFMD surveillance network was established in 2008 [32] and has historically focused on EV-A71 and CV-A16, as these serotypes cause most severe and fatal HFMD cases. EV-B serotypes were not systematically investigated as causative agents of HFMD until approximately 2010-2013 [72-73], with earlier surveillance focusing almost exclusively on EV-A71 and other EV-A serotypes. Furthermore, enterovirus surveillance in Guizhou has concentrated mainly on AFP (especially poliovirus) and HFMD cases associated with EV-A71 and other EV-A serotypes. Even after broader EV-B monitoring was implemented, E-3 has not been associated with severe HFMD outbreaks, leading to long-term underdetection. Consequently, the relatively low detection rate of E-3 in HFMD patients is likely due to inadequate targeted surveillance. Consistent with limited surveillance, the first nearly full-length E-3 genomes from HFMD patients were reported only in 2022 in Yunnan Province, China [51], and no severe cases of E-3-associated HFMD have been documented globally to date.

References are as follows:

1. Zhang T, Du J, Xue Y, Su H, Yang F, Jin Q. Epidemics and Frequent Recombination within Species in Outbreaks of Human Enterovirus B-Associated Hand, Foot and Mouth Disease in Shandong China in 2010 and 2011. PLoS One **2013;**8:e67157.

2. Tian H, Zhang Y, Sun Q, Zhu S, Li X, Pan Z, Xu W, Xu B. Prevalence of multiple enteroviruses associated with hand, foot, and mouth disease in Shijiazhuang City, Hebei province, China: outbreaks of coxsackieviruses a10 and b3. PLoS One **2014;**9:e84233.

3. Li Y, Chang Z, Wu P, Liao Q, Liu F, Zheng Y, Luo L, Zhou Y, Chen Q, Yu S, Guo C, Chen Z, Long L, Zhao S, Yang B, Yu H, Cowling BJ. Emerging Enteroviruses Causing Hand, Foot and Mouth Disease, China, 2010-2016. Emerg Infect Dis **2018;**24:1902-1906.

17. Comment

Page 16, line 450. Please, check and revise "mild and severe strains".

Answer:

We have revised the relevant statement in the manuscript (highlighted in blue; lines 505-507, page 18).

The relevant statement in the manuscript is as follows:

Sequence analysis revealed distinct genetic diversity between E-3 strains from the severe and mild case groups, particularly in the UTRs, 2A, 2C, 3A, and 3D regions, suggesting the accumulation of specific mutations during their divergence from a common ancestor.

18. Comment

Page 16, line 451. About the statement "independent evolutionary pathways": the phylogenies show clearly that strains from mild and severe cases are "only" diverging from one another while they derive from a single evolutionary pathway.

Answer:

We have revised the relevant statement in the manuscript (highlighted in blue; lines 505-507, page 18).

The relevant statement in the manuscript is as follows:

Sequence analysis revealed distinct genetic diversity between E-3 strains from the severe and mild case groups, particularly in the UTRs, 2A, 2C, 3A, and 3D regions, suggesting the accumulation of specific mutations during their divergence from a common ancestor.

19. Comment

Line 462. About the statement "This study elucidates the relationship between genomic variation and pathogenicity", I would suggest something like "This study provides avenues for further investigation into the relationship between observed genetic variations and the pathogenicity of E-3 strains."

Answer:

We have revised the relevant statement in the manuscript (highlighted in blue; lines 517-519, page 18).

The relevant statement in the manuscript is as follows:

This study provides avenues for further investigation into the relationship between observed genetic variations and the pathogenicity of E-3 strains.

Best wishes.

Shijun Li, PhD

E-mail: zjumedjun@163.com

April 19, 2026

Re: Spectrum03136-25R2 (Genetic Variation, Recombinant Characteristics, and Seroprevalence Analysis of Echovirus 3 Causing Severe and Mild Cases of Hand, Foot, and Mouth Disease in Guizhou Province)

Dear Dr. Shijun Li:

Your manuscript has been accepted, and I am forwarding it to the ASM production staff for publication. Your paper will first be checked to make sure all elements meet the technical requirements. ASM staff will contact you if anything needs to be revised before copyediting and production can begin. Otherwise, you will be notified when your proofs are ready to be viewed.

Data Availability: ASM policy requires that data be available to the public upon online posting of the article, so please verify all links to sequence records, if present, and make sure that each number retrieves the full record of the data. If a new accession number is not linked or a link is broken, provide production staff with the correct URL for the record. If the accession numbers for new data are not publicly accessible before the expected online posting of the article, publication may be delayed; please contact ASM production staff immediately with the expected release date.

Publication Fees: For information on publication fees and which article types have charges, please visit our website. We have partnered with Copyright Clearance Center (CCC) to collect author charges. If fees apply to your paper, you will receive a message from no-reply@copyright.com with further instructions. For questions related to paying charges through RightsLink, please contact CCC at ASM_Support@copyright.com or toll free at +1-877-622-5543. CCC makes every attempt to respond to all emails within 24 hours.

ASM Membership: Corresponding authors may join or renew ASM membership to obtain discounts on publication fees. Need to upgrade your membership level? Please contact Customer Service at Service@asmusa.org.

Embargo Policy: A press release may be issued as soon as the manuscript is posted on the Spectrum Latest Articles webpage. The corresponding author will receive an email with the subject line "ASM Journals Author Services Notification" when the article is available online.

The ASM Journals program strives for constant improvement in our submission and publication process. Please tell us how we can improve your experience by taking this quick Author Survey.

Thank you for submitting your paper to Spectrum.

Sincerely,
Clinton Jones
Editor
Microbiology Spectrum

Reviewer #1 (Comments for the Author):

In a previous review, I noted that as the 4 strains from patients with less severe disease are highly related and so are the 4 strains from patients with severe disease, it will be difficult to find sites which reflect actual virulence with so few isolates. However, the authors have provided data comparing cellular infection contrasting the mild and severe strains which suggests variations differ in characteristics of basic virus replication in cells. I note that the authors have addressed the criticism of previous reviews to the effect that it is difficult to assess how virulent a virus is based on isolation from mild and severe cases due to host factors affecting the assignment to these groups in the discussion. With the analysis of the presence and consequences of these variations in similar sites in enteroviruses, the analysis of the emergence of variants associated with severe disease in echovirus 3 has been done very thoroughly.
The authors have extended and corrected the referencing in this manuscript. I did note on lines 158-159, the AO/EB method for staining for apoptosis should be referenced.

Reviewer #3 (Public repository details (Required)):

nucleotide sequences

Reviewer #3 (Comments for the Author):

I am satisfied with the authors' responses to the comments, with one exception: this is comment no. 11. It concerns the 3D structures of proteins P1, P2 and P3 and figure 4.
I will clarify my query, which may not have been expressed clearly. I would like to know how the authors validated the 3D structures obtained, in particular the structure of the virion capsid proteins (P1 sequence).
I have a specific concern regarding the 3D structure of proteins P2 and P3. These are polyproteins that do not represent functional proteins (except 3AB and 3CD). This is why studying the 3D structure of the whole P2 and P3 polyproteins is of no biological interest. Could you clarify your aims and the results.
