# Supplementary material for: Genetic variation, recombinant characteristics, and seroprevalence analysis of echovirus 3 causing severe and mild cases of hand, foot, and mouth disease in Guizhou Province
Source: Microbiol Spectr. 2026 Jul 2;14(8):e03136-25. doi: 10.1128/spectrum.03136-25 (PMC13436393; doi:10.1128/spectrum.03136-25)
Supplement: Supplementary file 2 [file spectrum.03136-25-s0001.docx]

**Fig. S1** The phylogenetic trees are based on the VP1, P1, P2, and P3 sequences of E-3. The four strains from severe cases are indicated by red solid circles, the four strains from mild cases are indicated by blue triangles, and the E-3 prototype is indicated by a brown diamond. Eight Guizhou E-3 strains and other E-3 strains were analyzed through nucleotide alignment using the neighbor-joining method and the Kimura 2-parameter model implemented in MEGA 11.0. Bootstrap values >75 are shown and inferred from bootstrapping with 1,000 replicates. (A) VP1 coding sequences; (B) P1 coding sequences; (C) P2 coding sequences; (D) P3 coding sequences.
